# Conserved and specific features of *Streptococcus pyogenes* and *Streptococcus agalactiae* transcriptional landscapes

**DOI:** 10.1186/s12864-019-5613-5

**Published:** 2019-03-22

**Authors:** Isabelle Rosinski-Chupin, Elisabeth Sauvage, Agnès Fouet, Claire Poyart, Philippe Glaser

**Affiliations:** 1 Ecology and Evolution of Resistance to Antibiotics, Institut Pasteur-APHP-Université Paris Saclay, UMR3525 CNRS, Paris, France; 2 INSERM U1016, Institut Cochin, CNRS UMR 8104, Université Paris Descartes (UMR-S1016), Paris, France; 3 Centre Nationale de Référence des Streptocoques, Hôpitaux Universitaires Paris Centre, Cochin, Assistance Publique Hôpitaux de Paris, Paris, France

**Keywords:** Regulatory RNAs, 5′ UTRs, Promoters, Operons, Regulatory network evolution, Antisense transcription

## Abstract

**Background:**

The human pathogen *Streptococcus pyogenes*, or group A *Streptococcus*, is responsible for mild infections to life-threatening diseases. To facilitate the characterization of regulatory networks involved in the adaptation of this pathogen to its different environments and their evolution, we have determined the primary transcriptome of a serotype M1 *S. pyogenes* strain at single-nucleotide resolution and compared it with that of *Streptococcus agalactiae,* also from the pyogenic group of streptococci.

**Results:**

By using a combination of differential RNA-sequencing and oriented RNA-sequencing we have identified 892 transcription start sites (TSS) and 885 promoters in the *S. pyogenes* M1 strain S119. 8.6% of *S. pyogenes* mRNAs were leaderless, among which 81% were also classified as leaderless in *S. agalactiae*. 26% of *S. pyogenes* transcript 5′ untranslated regions (UTRs) were longer than 60 nt. Conservation of long 5′ UTRs with *S. agalactiae* allowed us to predict new potential regulatory sequences. In addition, based on the mapping of 643 transcript ends in the *S. pyogenes* strain S119, we constructed an operon map of 401 monocistrons and 349 operons covering 81.5% of the genome. One hundred fifty-six operons and 254 monocistrons retained the same organization, despite multiple genomic reorganizations between *S. pyogenes* and *S. agalactiae*. Genomic reorganization was found to more often go along with variable promoter sequences and 5′ UTR lengths. Finally, we identified 117 putative regulatory RNAs, among which nine were regulated in response to magnesium concentration.

**Conclusions:**

Our data provide insights into transcriptome evolution in pyogenic streptococci and will facilitate the analysis of genetic polymorphisms identified by comparative genomics in *S. pyogenes.*

**Electronic supplementary material:**

The online version of this article (10.1186/s12864-019-5613-5) contains supplementary material, which is available to authorized users.

## Background

*Streptococcus pyogenes* or Group A *Streptococcus* (GAS) is a Gram-positive human-restricted pathogen responsible for a broad range of mild to severe diseases such as pharyngitis, impetigo, bacteremia, necrotizing fasciitis, streptococcal toxic shock and for post-infectious complications such as acute rheumatic fever or glomerulonephritis. It is estimated that GAS accounts for 600 to 700 million infections globally per year among which about 18 millions are considered as severe and lead to over 500.000 deaths annually. GAS strains are classified based on the amino-terminal sequence of the variable cell-surface M protein, encoded by the *emm* gene and more than 200 *emm* (M) types have been recorded. While rates of severe GAS infections decreased at the beginning of the twentieth century especially in industrialized countries, there was a re-emergence of invasive infections during the late 1980s [1]. This re-emergence was mainly driven by changes in the M types circulating in Europe with an increase in infections caused by M1 and M3 GAS [2].

Sequencing of thousands of GAS genomes has shed light on the evolutionary forces that accounted for the expansion of more virulent clones. In particular increased pathogenicity of M1 clone was found to be linked to the acquisition of new virulence genes such as genes encoding DNase D2 (Sda2), streptococcal pyrogenic exotoxin A superantigen (SpeA), NAD + -glycohydrolase and streptolysin O [3, 4]. Modifications in the regulatory networks controlling expression of proteins involved in host interaction and virulence were also shown as major contributors to the increased pathogenicity of M1, M3 and M89 strains [5–9]. These modifications included variations in regulatory gene sequences as well as polymorphisms in promoter regions of genes encoding virulence factors. For instance, in M3 strains, evidence for strong diversifying selection was observed in the coding regions of the master regulator of virulence (CovRS) two-component system and of the regulator of protease B RopB [9].

Over the last 10 years RNA-sequencing (RNA-seq) has offered tremendous power for high-resolution transcriptome characterization allowing both differential expression analysis and single nucleotide mapping of transcript ends. While the *Streptococcus* genus contains many pathogenic species, data on genome-wide transcriptome organization of streptococcal species are scarce and have only been obtained for *Streptococcus agalactiae*, *Streptococcus suis* and recently *Streptococcus pneumoniae* [10–12]. We previously combined differential RNA-sequencing (dRNA-seq) and strand-specific RNA-sequencing (RNA-seq) to establish a comprehensive map of *S. agalactiae* transcriptome, providing information on promoters, operon structure and non-coding RNAs and revealing new regulatory mechanisms in this species [10]. Although *S. pyogenes* belongs to the same group of streptococci, the pyogenic group, as *S. agalactiae*, the two species are phylogenetically distant suggesting that their transcriptional organization may have substantially diverged. In *S. pyogenes*, a systematic identification of transcript 5′ and 3′ ends is still lacking. Non-coding RNAs (ncRNAs) have been characterized by using bioinformatics, microarrays and more recently RNA-seq [13–20]. This led to the description of hundreds of ncRNAs with limited overlaps between studies. Only a handful of ncRNAs, such as FasX, Pel, RivX or MarS have been functionally characterized [21–24].

To fill this knowledge gap for this critical pathogen, we carried out the genome-wide determination of transcriptional start sites (TSS) and characterized promoter regions and 5′ UTR. We combined this approach to strand-specific RNA-seq to determine operon organization and ncRNAs and compared these primary transcriptome data with those we established in *S. agalactiae* to identify regulatory mechanisms under conservative selection in pyogenic streptococci and evolutionary adaptations specific to *S. pyogenes*. Our findings contribute to a better understanding of the complex gene regulation in this species, while providing insights into transcriptome evolution in streptococci.

## Results

### Genome-wide identification of *S. pyogenes* transcription start sites and promoters

We selected an invasive M1 strain (S119) isolated from a blood culture in 2008 in France. Its complete genome sequence was determined and annotated. It differs from that of the M1 epidemic strain MGAS5005 (NC_007297.2) by 65 single-nucleotide polymorphisms (SNP) and 14 indels of 1 to 21 nucleotides (nt). Compared to MGAS5005, S119 has a wild-type *covS* kinase gene and carries an additional prophage 98.7% identical over 93.7% of its sequence with prophage S370.1 of the M1 strain SF370 and similarly inserted in the first codons of *pepD* encoding a dipeptidase.

We first determined the TSSs genome-wide map of strain S119 by differential RNA-seq (dRNA-seq) based on selective Tobacco Acid Pyrophosphatase (TAP) treatment and 5′ adapter ligation to differentiate primary transcripts (5′ tri-phosphate) and processed RNAs (5′ mono-phosphate) [10, 25]. Strain S119 was grown to late exponential phase in Todd-Hewitt broth with yeast extract (THY) supplemented or not with 15 mM MgCl_2_, as Mg^2+^ cations modify gene expression in *S. pyogenes* mostly by activating the CovS kinase and this effect was found to be maximum at late exponential phase [26]. RNA prepared from both experimental conditions were mixed and a total of ~ 45 M reads under TAP+ and TAP- conditions were obtained (See Additional file 1: Table S1). Reads were aligned on *S. pyogenes* S119 genome sequence, with 5.4 and 2.3 million reads aligning to non-ribosomal regions under TAP+ and TAP- conditions respectively. The number of reads beginning at each base was compared under both conditions. The statistical assignment of TSS was completed by visual identification of TSS for genes with low expression levels using both TAP+ and TAP- reads and analysis of 100 million supplementary reads generated by whole transcript strand-specific RNA-seq experiments and obtained under the same growth conditions. In total, we determined 892 TSS (See Additional file 2: Table S2).

**Fig. 1:**
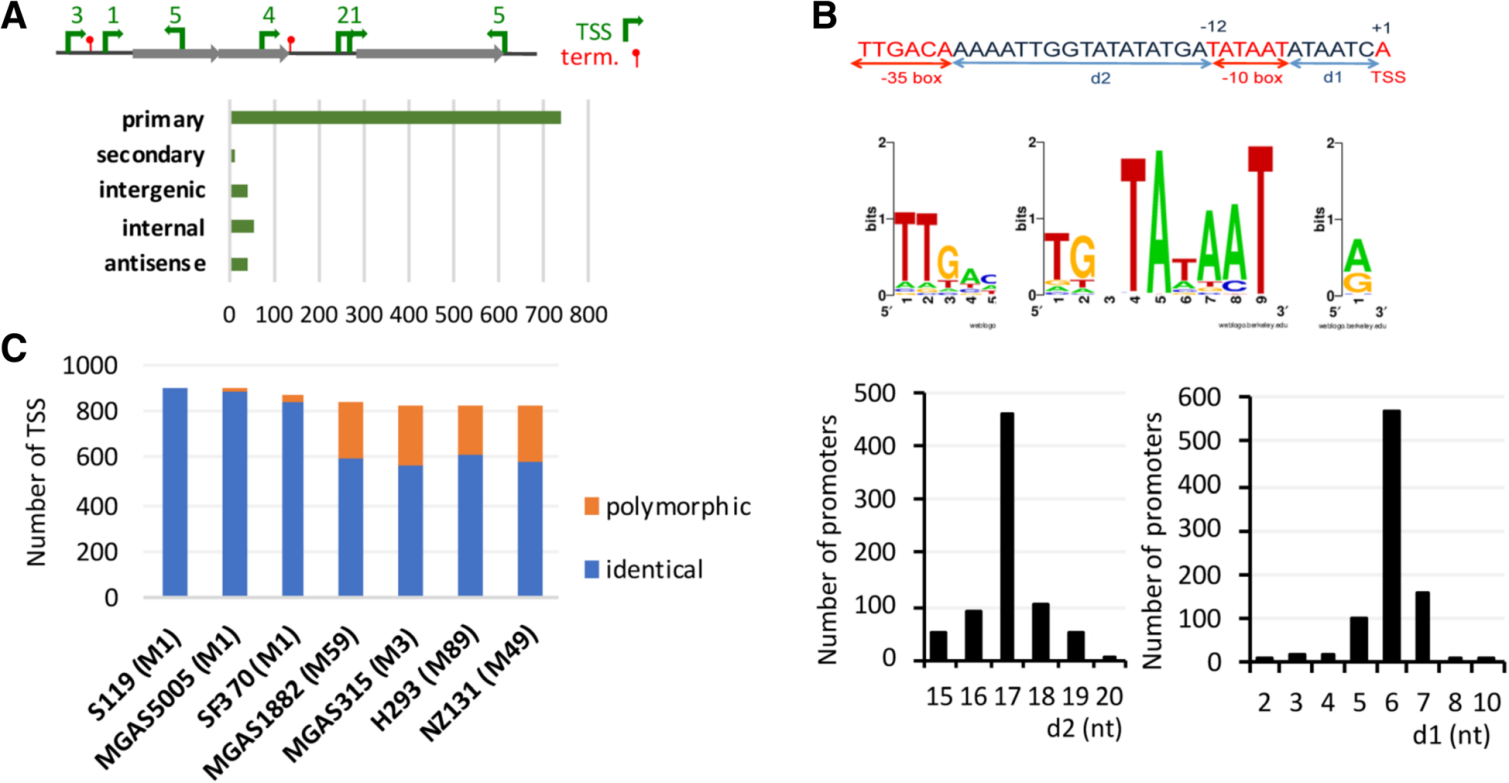
Characteristics of transcription start sites in *S. pyogenes.* **a** Classification of TSS according to their position relative to CDS; **b** Consensus sequence for *S. pyogenes* promoters: motif search reveals extended Pribnow or − 10 boxes and less conserved − 35 boxes, typical of sigma70 dependent promoters, upstream of 885/892 *S. pyogenes* TSSs. The consensus sequence was generated by using WebLogo (http://weblogo.berkeley.edu/logo.cgi). The mean distances between the − 10 box and the TSS (d1) and between the − 10 and − 35 boxes (d2) were calculated between the 3′ end nucleotide of the − 10 box and the TSS and between the 3′ nucleotide of the − 35 box and the 5′ nucleotide of the − 10 sequence; **c** Comparison of the 50 nt long sequences upstream of TSS between strains belonging to different emm lineages (the emm type is indicated between brackets). The number of 100% identical sequences is indicated by blue bars while sequences showing polymorphisms are indicated by orange bars

TSS were annotated according to their position relative to CDSs (Fig. 1a). 85% (*n* = 755) were located upstream of protein-coding genes, among which 741 corresponded to primary TSS and 14 to secondary TSS. Seventy-seven TSS were located inside CDS, 53 initiating transcription in the same orientation as the CDS and 24 in the opposite orientation. Nineteeen supplementary TSS, close to CDS (< 250 nt) also initiated an antisense transcription. Finally 41 TSS were characterized in intergenic regions and were upstream of potential ncRNA (28) and tRNA (13) genes (See Additional file 2: Table S2).

We predicted promoter sequences upstream of all but seven identified TSSs (See Additional file 2: Table S2). All but two were similar to the consensus of the housekeeping Sigma70 binding promoters (TTGACA-X_15/21bp_-TATAAT) [27]. A -10 box was found for all of them while a − 35 box was predicted in 85% (*n* = 753) (Fig. 1b). In addition to Sigma70, GAS expresses two additional sigma factors, SigX1 and SigX2. These two sigma factors were shown to be expressed at very low levels in THY [28]. Accordingly, we only detected two TSS located downstream potential SigX binding sites (TACGAATA). These TSS were associated with low-abundance transcripts for genes *SP119_0098* encoding a single-strand binding protein and *SP119_1410* encoding a paratox protein.

The first transcribed nucleotide was A (59%, *n* = 523) or G (36%, *n* = 323) reflecting the preference of the RNA polymerase for purine residues as initiator nucleotides, as observed in *Bacillus subtilis*, *S. agalactiae, S. pneumoniae* and *Escherichia coli* [10, 12, 27, 29]. TSSs were located at 11–13 nt from the predicted − 10 box in 93% (*n* = 828) of the cases (Fig. 1b). Shorter distance to the promoter was most often due to reiterative transcription which modified the apparent TSS position. We recently showed that it is frequent in *S. agalactiae*, affecting up to 15% of the TSSs [10]. By analyzing pseudo-templated nucleotides at transcript 5′ ends, we predicted 113 TSS (12.7%) with reiterated transcription in strain S119 (See Additional file 2: Table S2). The non-templated nucleotides were generally associated with nucleotide stretches on the DNA template and were most often A repeats (78%) (See Additional file 3: Figure S1). Like in *B. subtilis* and *S. agalactiae*, a reiterative incorporation of G nucleotides occurs at the TSS of *pyrG*, encoding the CTP synthetase and may regulate its transcription by attenuation during starvation for pyrimidine [10, 30].

We next searched for conservation of the TSS and the promoter sequence in the genome sequences of six strains belonging to five M types: M1 strains SF370 and MGAS5005, M3 strain MGAS315, M59 strain MGAS1882, M49 strain NZ131 and M89 strain H293. 89% (*n* = 794) of the promoters were conserved in all six strains (See Additional file 4: Table S3). The remaining 11% were lacking in one or several strains and belong to the variable genome of the strain: 44 were in prophage sequences and 49 were in small islands of polymorphism such as pilus loci. SNP in the 50 nucleotides preceding the TSS compared to strain S119 were observed in two (M1 strain MGAS5005) to 246 (M3 strain MGAS315) sequences (Fig. 1c). These polymorphisms may account for differences in transcription and physiological properties among GAS strains.

### Characterization of *S. pyogenes* transcriptional organization

To further describe the operon organization in *S. pyogenes* strain S119, we annotated transcript 3′ ends by using the strand-specific RNA-seq data. 65% of the CDS (*n* = 1192) were detected above a 3 RPKM (Reads Per Kilobase Million) threshold that reflected a continuous coverage along the CDS and allowed a characterization of the transcript 3′ ends. We detected 556 transcript ends (Additional file 5: Table S4) associated with these CDS and 87 supplementary 3′ ends associated with potential ncRNAs. Five hundred and seventy 3′ ends corresponded to rho-independent terminators predicted in silico [31–33]. These data were combined with the TSS data to draw an operon map of S119 (Additional file 6: Table S5). In total we predicted 401 monocistrons and 349 operons composed of 2 to 23 genes. Sixty operons were defined as composite as they encompass either internal promoters or internal terminators, which might lead to alternate transcriptional units and differential gene expression within the operon. This operon map covered 1506 CDS (81.5% of the total genome).

### *S. pyogenes* non-coding RNAs

Forty three TSS potentially initiated an antisense transcript and 29 TSS, including two TSS upstream of the CRISPR tracRNA, were in intergenic regions not directly linked to a protein coding gene. In addition, 27 5′ UTR of coding genes were predicted to contain an internal rho-independent terminator, potentially leading to the production of a ncRNA. The visual inspection of RNA-seq data allowed to determine the putative 3′ ends of 96 out of these 98 ncRNAs and to identify 19 supplementary ncRNAs. 11 of these 19 ncRNAs lacked a characterized TSS, and 8 were in the 5′ UTR of a coding gene missing a predicted rho-independent terminator. These ncRNAs might have been generated through cleavages of longer RNAs by one of the many nucleases involved in RNA processing and maturation. For instance, RNAse III dependent cleavages of the 5′ UTR of the *pnp* gene, encoding the polyribonucleotide phosphorylase, are predicted to produce two 22 and 30 nt-long ncRNAs [34] (Additional file 7: Figure S2). While the 30 nt-long ncRNA corresponding to the top of the step-loop structure was likely degraded, the 22 nt-long was detected in the RNA-seq experiment, which could indicate its stabilization through the formation of a duplex structure with *pnp* mRNA. Such a structure was found in *E. coli* to be a substrate for the polynucleotide phosphorylase, resulting in the retro-regulation of PNPase expression [35]. Our *S. pyogenes* data suggest a similar regulatory mechanism of the *pnp* gene in a Gram-positive bacterium. In total, we identified 117 putative regulatory RNAs (Table 1 and Additional file 8: Table S6). Thirty six antisense ncRNAs were novel including seven longer than 1000 nucleotides considered as long antisense RNAs (lasRNAs).

**Table 1:** Characteristics of sRNAs detected in the study

Position relative to proximal CDS	Number	Shared by *S. agalactiae*	Novel	With Rfam annotation or well characterized	TSS characterized	Length > 1000 nt	low expression
3′ UTR:	6	0	1	1 (SSRC30)	NA^a^	–	–
Antisense:	45	2	35	0	43	7	15
5′ UTR:	36	24	2	20 (1Lacto-rpoB; Riboswitches: 1 FMN, 1TPP,1 Gly, 1 yybp-ykoY, 1 purine;7 T-box; ribosomal leaders: 23S-methyl, L-20 leader, L-10 leader, L21-leader; 2 pyrR)	35	–	1
Intergenic:	30	10	8	12 (SRP, tmRNA, 6S, RNAseP,CRISPR1, tracRNA,CRISPR2, FasX, pel, asd, csRNA15,csRNA25)	28	–	4

^a^Not applicable

**Fig. 2:**
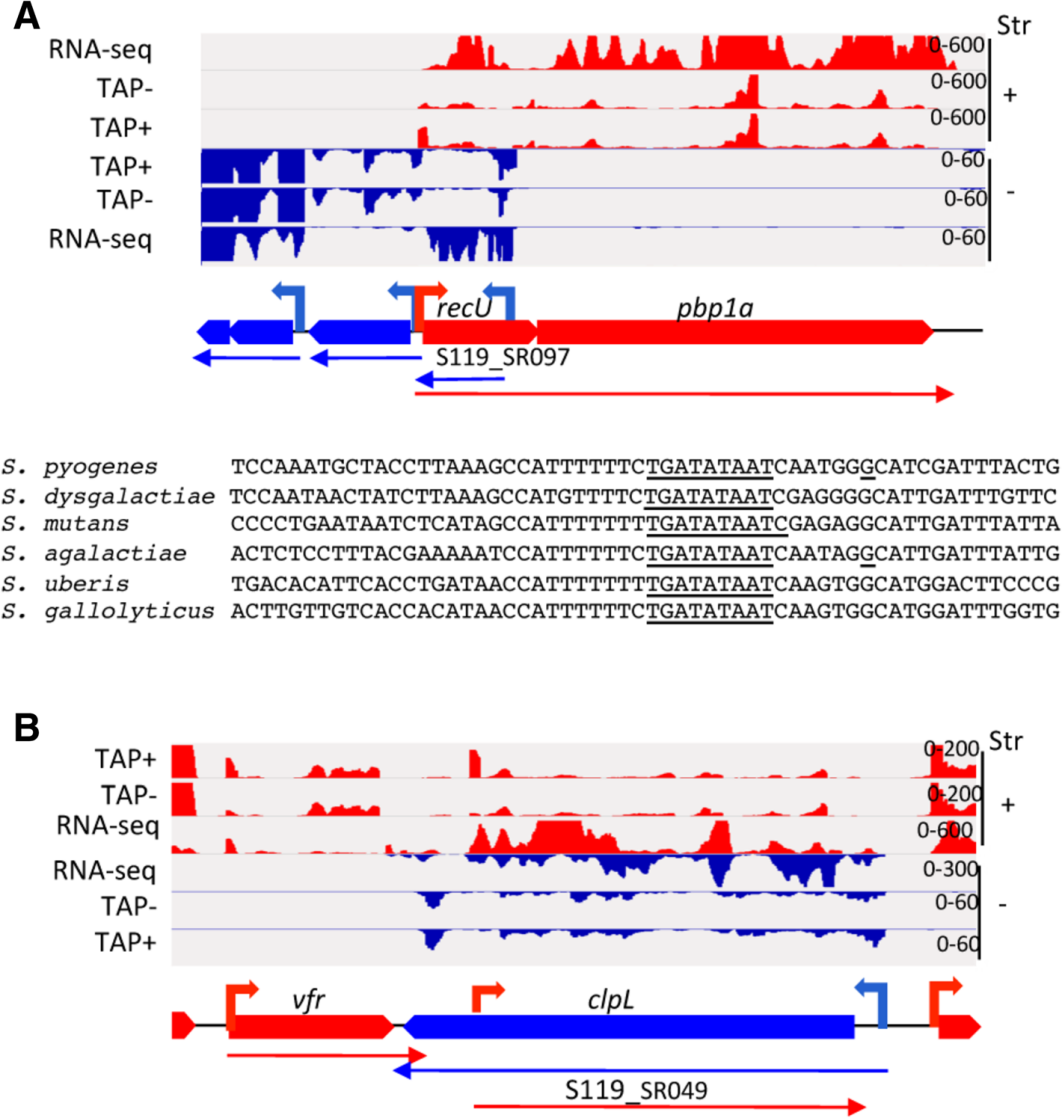
Detection of antisense ncRNAs and mapping of their TSS. **a** Detection of SP119_SR097 antisense to *recU* and sequence alignment showing the conservation of the corresponding promoter among streptococci (the extended − 10 box sequence and the TSS that have been characterized are underlined). **b** Detection of SP119_SR049 potentially regulating expression of *clpL* in *S. pyogenes*. The sequence reads mapped to the genome of strain S119, in conditions of dRNA-seq: strand-specific sequencing of transcript 5′ ends with (TAP+) and without (TAP-) TAP treatment, and strand-specific RNA-seq, are visualized by using IGV. On the schematic view of the gene organization, the protein coding genes annotated on the (+) and (−) strands (Str) are indicated by red and blue large arrows respectively. TSSs are depicted as small arrows (red and blue for plus and minus strands respectively). The identified transcripts are shown by thin arrows

Interestingly, like in *S. agalactiae,* an antisense RNA overlapped the 3′ quarter of *recU* CDS (*SP119_1350*) required for chromosome segregation and DNA repair (Fig. 2a). In various Firmicutes *rec*U is transcribed as an operon upstream of the gene coding for PBP1a (named PBP2 in *S. aureus*). While in *S. aureus*, a second promoter, internal to *recU* directs a *recU* independent expression of PBP2 [36], such a promoter is found neither in *S. agalactiae* nor in *S. pyogenes*. We propose that, instead, the *recU* antisense allows the differential expression of *recU* and *pbp1a* in streptococci.

**Fig. 3:**
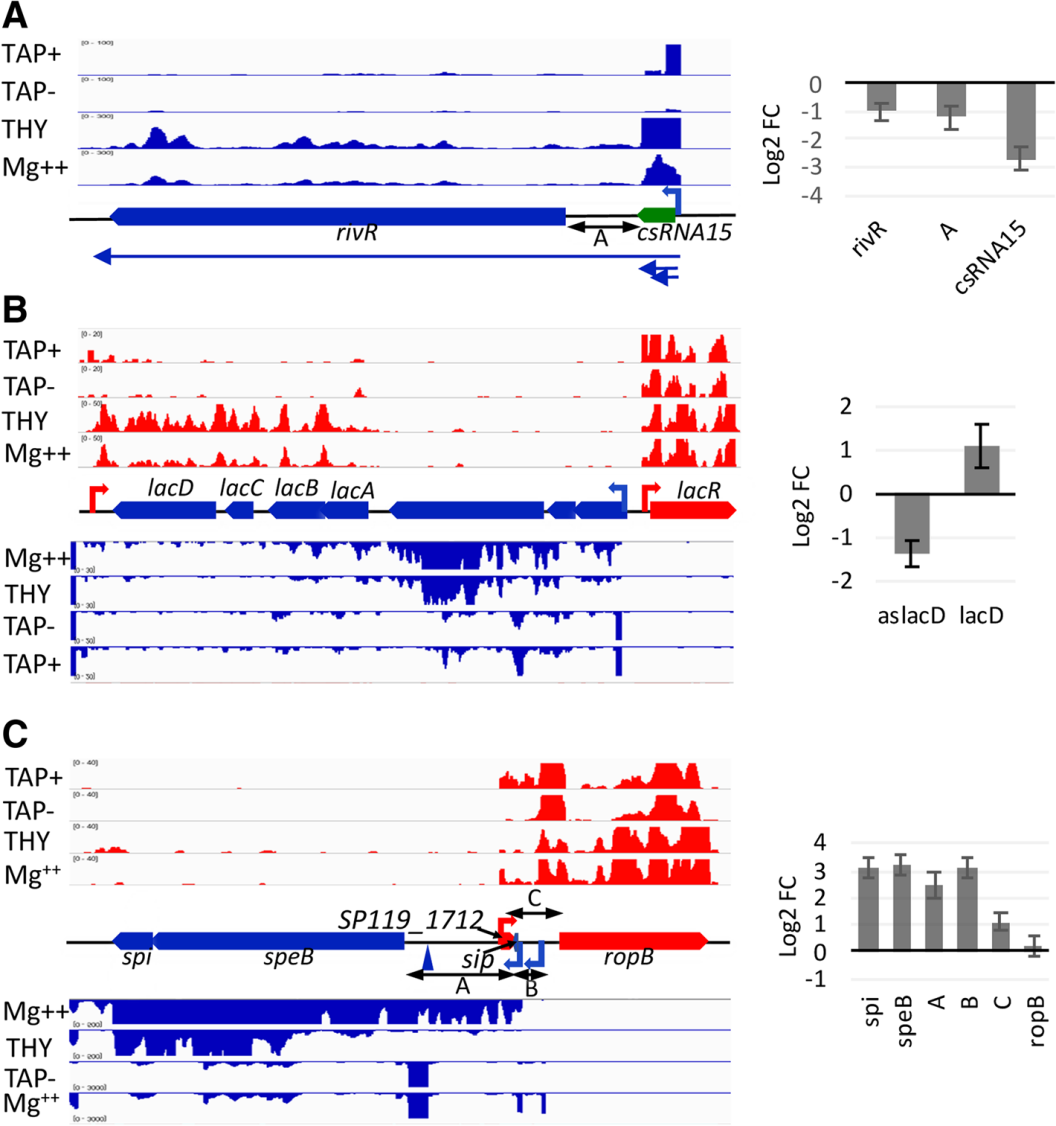
Variations in strain S119 transcriptome in response to high Mg^2+^ concentrations. Left: IGV captures of RNA-seq coverages in conditions of late exponential growth phase in THY broth supplemented (Mg^2+^) or not (THY) with 15 mM MgCl_2_ and TAP^+^/TAP^−^ results of dRNA-seq experiments, shown on the (+) and (−) strands and schematic views of TU organizations. Right: Log_2_ values of the expression fold-changes (FC) between samples prepared at high versus low Mg^2+^ concentrations. Mean value±SEM (*N* = 3). **a**. Down-regulation of csRNA15-*rivR* TU in conditions of high Mg^2+^ concentration; **b** Inverse regulation of *pts-lacABCD*.1 operon and of the *lacABCD* antisense transcript, in response to variations in Mg2^+^ concentration. **c** Up-regulation of the *speB-spi* operon at high Mg^2+^ concentrations; the two TSS at − 696 and − 841 relative to the translation initiation codon for *speB* are indicated by thin arrows while a major cleavage site located at − 137 and associated with a TAP+/TAP- ratio close to 1 is shown by a blue triangle. The *speB-spi* transcript 5′ UTR overlapped the 5′ UTR of the *rop*B transcript, whose TSS was characterized 368 nt upstream of *ropB* translation initiation codon. The CDS encoding the recently described peptide SIP [64] from the *speB-spi* 5’UTR is indicated. A potential initiation codon is also present three nucleotides after the *rop*B TSS, a configuration reminiscent of leaderless RNAs that would give rise to a 26 amino-acid peptide we annotated as *SP119_1712*. Differential expression analysis of the 5’UTR associated with the two *speB*-*spi* TSS showed that both promoters were affected by Mg^++^ status. While no increase in *ropB* expression was detected over the CDS, a two-fold increase in the coverage of *SP119_1712* was observed in Mg-rich medium, suggesting a supplementary level of regulation on *ropB*

We detected a lasRNA complementary to the *lacABCD* genes (*SP119_1392–95)* which could be involved in the regulation of the LacD.1 aldolase, described as a metabolic sensor negatively regulating SpeB expression [37] (Fig. 3b). Antisense transcription was also exceeding sense transcription of *clpL* (*SP119_0711*) encoding a chaperone protein (Fig. 2b)*. clpL* and the virulence factor related (*vfr*) gene form convergent transcription units. Therefore, *clpL* antisense transcript might ensure that *clpL* is minimally expressed under current growth conditions and does not interfere with *vfr* transcript level.

In total, 19 out the 81 intergenic and antisense ncRNAs were encoded in prophages, illustrating the major role of ncRNAs in phage regulation. In particular we discovered a new family of antisense sRNAs: SP119_SR034, SP119_SR071, SP119_SR086, SP119_SR106 similarly located in the intergenic regions upstream of the integrase genes of prophages SP119-P1, −P2, −P3 and -P4 (See Additional file 9: Figure S3). These ncRNAs might contribute to the silencing of integrase expression during the lysogenic phase.

Finally, differential expression analysis of the RNA-seq data revealed that expression of nine ncRNAs was modified in culture medium containing high Mg^2+^ concentration (fold-change > 2 and FDR ≤ 0.05) (see Additional file 10: Table S7). These included the sag/pel sRNA, the SP119_SR120 sRNA located upstream of the *scpA-fba* operon, the csRNA15/ SP119_SR011 located upstream of *rivR* (Fig. 3a), the csRNA25/SP119_SR122 as well as the ncRNA antisense to the *lacDCBA.1* genes (Fig. 3b). This suggests that ncRNAs are involved in the regulatory networks leading to Mg^2+^ mediated expression modifications. In addition we also detected expression modifications of 161 coding genes (50 down-regulated and 111 up-regulated) in the Mg^2+^ supplemented medium (see Additional file 10: Table S7). In agreement with published data the down-regulated genes included several genes coding for virulence factors. The expression of the gene encoding the Mga transcriptional regulator and of known direct targets (*sclA*, *scpA* and *fba)* [38, 39] was also down-regulated. There was an up-regulation of the gene coding for the immunoglobulin G binding protein Grab, of the genes involved in the synthesis of the pilus (*SP119_0103–0107*) and of the gene encoding the laminin-binding protein Lmb. In addition, we observed a ~ 9 fold up-regulation of the operon coding for the Streptococcal pyrogenic exotoxin B (*SpeB*) and the inhibitor of protease activity Spi under high Mg^2+^ concentrations in the M1 S119 strain (Fig. 3c). Differential expression analysis of the 5′ UTR associated with the two *speB* TSS (this work and [40, 41]) showed that both promoters were likely affected by Mg^2+^ status. These 5′ UTR overlap in antisense orientation the 5′ end of the *rop*B transcript. A potential translation initiation codon ATG is present three nucleotides after *ropB* TSS, a configuration reminiscent of leaderless RNAs, that would give rise to a 26 amino-acid long peptide we annotated as SP119_1712. While no increase in *ropB* expression was detected over *rop*B CDS, a two-fold up-regulation of *SP119_1712*-*ropB* 5′ UTR was observed under high Mg^2+^ concentration. The decoupling between *SP119_1712* and *ropB* expression suggests that supplementary regulation occurs either at transcription termination on the intergenic region between *SP119_1712* and *ropB* or at the level of transcript stability.

### Comparison of *S. pyogenes* and *S. agalactiae* transcriptional organizations

**Fig. 4:**
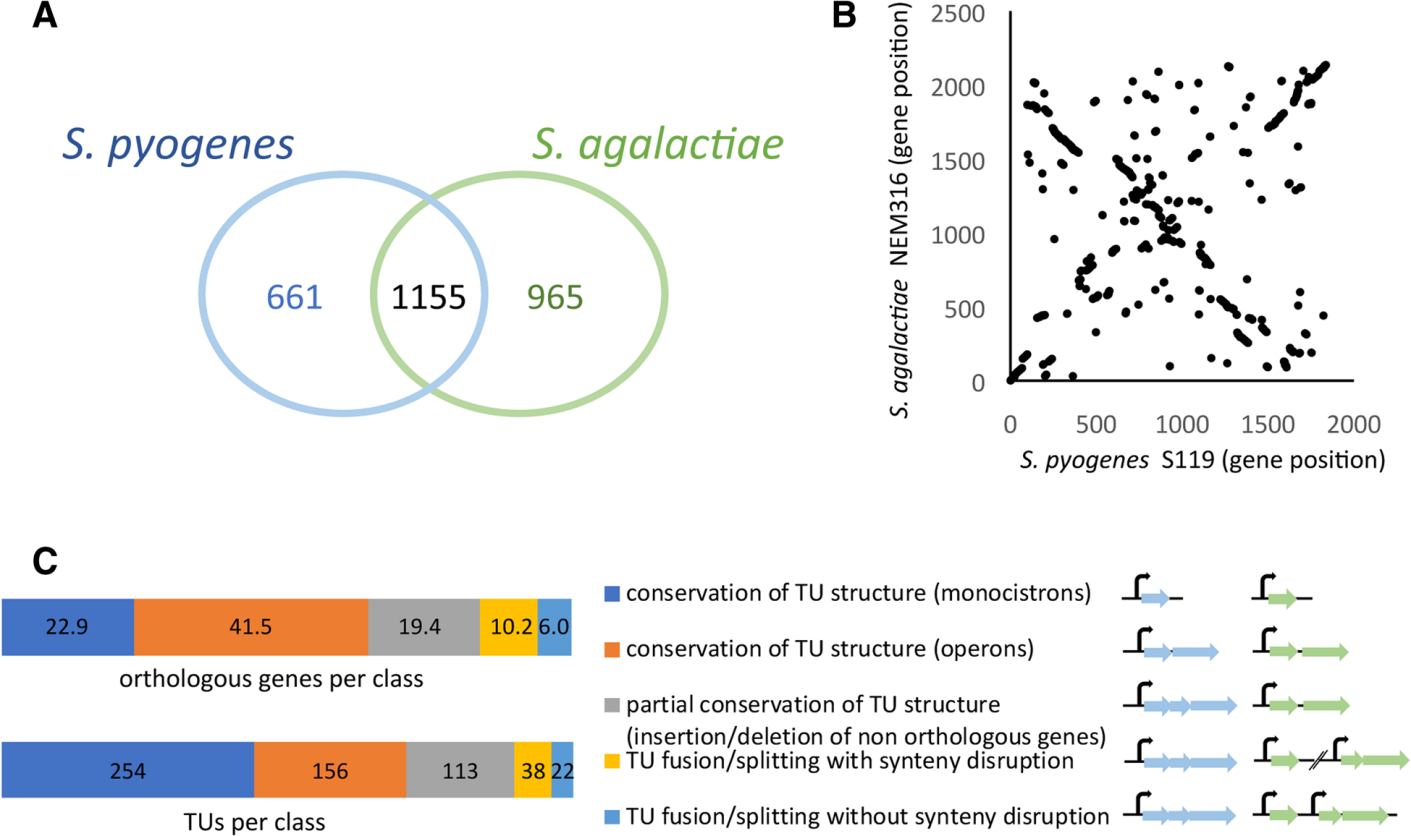
Comparison of *S. pyogenes* and *S. agalactiae* transcriptional architectures. **a**. Venn diagram showing orthologous and species-specific genes between *S. pyogenes* strain S119 and *S. agalactiae* strain NEM316. **b** Scatter plot of gene order-based synteny comparison between both species. **c**. Conservation of TU structure among both species expressed as the percentage of genes in each category, defined according to the figure keys, relative to the total number of orthologs (upper diagram) and as the number of TUs in each category (lower diagram)

While belonging both to the pyogenic group, *S. pyogenes* and *S. agalactiae* are phylogenetically distant with an evolutionary distance of 0.21 amino acid substitutions per site based on the sequence of 136 genes belonging to *Streptococcus* core genome. Reciprocal best BlastP alignments between *S. pyogenes* S119 and *S. agalactiae* NEM316 proteome allowed to predict 1155 orthologous genes (Fig. 4a). Comparison of the gene order showed blocks of synteny of various lengths and genome reshufflings (Fig. 4b), with gene insertions/deletions and gene re-localizations. Comparing the number of primary TSS relative to the total number of CDS in *S. pyogenes* and in *S. agalactiae* revealed similar proportions in both species with respectively 40.8 and 42% of coding genes preceded by a primary TSS respectively (Table 2).

**Table 2:** Comparison of the main characteristics of TSS mapped in *S. pyogenes* and *S. agalactiae*

	*S. agalactiae* NEM316	*S. pyogenes* S119	
Genome length	2.21 Mb	1.88 Mb	
Number of coding genes (including pseudogenes)	2120	1816	
Number of species-specific coding genes	965	661	
Total number of TSS	1210	892	
Primary TSS	891	741	
Coding genes preceded by a primary TSS	42.0% (891/2120)	40.8% (741/1816)	
Secondary TSS	36	14	
Internal TSS at less than 200 nt from next CDS in similar orientation	26	11	
Other internal TSS in sense orientation	165	42	
TSS for antisense RNAs	39	43	
Intergenic orphan TSS	53	41	
	in both species	only in GBS	only in GAS
orthologous genes with a primary TSS	533	45	39
orthologous genes with a secondary TSS	2	21	9
orthologous genes with an internal promoter	14	92	32
species-specific gene with a primary TSS	–	313	169
species-specific gene with a secondary TSS	–	13	3
species-specific gene with an internal TSS	–	85	7

To determine how the genomic reorganization modified operon structure and created/eliminated promoter sequences, we compared the TSS maps and the operon organizations in *S. pyogenes* and in *S. agalactiae*. Among the 1155 shared genes, 1107 were attributed to transcriptional units (TU) expressed in both species in rich culture broth, allowing a comparison of their organization. Two hundred and fifty four genes (22.9%) were expressed as monocistrons in both species and 459 genes (41.5%) were expressed in operons (156 operons) of same size and composition (Fig. 4c)*.* In addition, 215 genes (19.4%) were expressed as partially conserved operons (113 operons) containing additional species-specific genes. Eventually only 16.2% of the shared genes belonged to TUs that were split in the other species. This modification of TU structure occurred in association with a breakdown of the synteny block in ~ 64% of the cases.

**Fig. 5:**
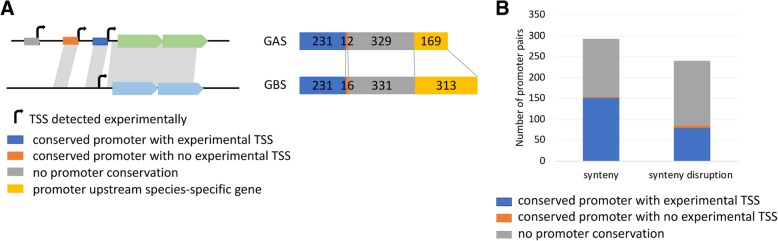
Conservation of promoter sequences between *S. pyogenes* and *S. agalactiae*. **a**. Schematic representation of the analysis and number of primary promoters in each category according to the figure keys in *S. pyogenes* (GAS) and *S. agalactiae* (GBS) genomes. The 50 nt upstream of each TSS were aligned against the 5′ UTR of the orthologous gene of the other species as described in Materials and methods. Sequences that showed an alignment were classified either as conserved promoter with experimental TSS or conserved promoter with no experimental TSS whether or not, respectively, their 3′ end corresponded to a TSS determined experimentally in the orthologous sequence. When sequence alignment was not significant, promoters were considered as not conserved. The number of promoters of species-specific genes is also indicated; **b** Number of promoters in each class depending on the conservation of synteny with the upstream gene

Then we looked at promoter conservation between both species. Among the 1155 shared genes, 617 were preceded by a primary TSS in at least one species: 533 (86%) were associated with a primary TSS in both species, 39 only in *S. pyogenes* and 45 only in *S. agalactiae* (Table 2*)*. In order to evaluate the conservation of promoters we compared the DNA sequence upstream of TSS of orthologous genes (Fig. 5a). For 37% (*n* = 231) of the 617 gene pairs, we predicted the promoters as conserved since a TSS was detected in both species and the 50 nt sequences upstream of these TSS could be aligned. For 6 gene pairs the promoters were different and for 6 GAS genes and 10 GBS genes the promoter sequence was present but not used in the other species. For the remaining genes the similarity between *S. pyogenes* and *S. agalactiae* upstream sequences was too low to conclude on promoter conservation. These promoters might have been subjected to evolutionary changes leading to species-specific regulations. Alternatively, they might correspond to poorly regulated promoters whose activity only requires the binding of the RNA-polymerase. Interestingly, genes maintaining synteny with their upstream neighbor showed a higher proportion of conserved promoters (47 versus 27%) (Fig. 5b) suggesting that recombination is associated with promoter sequences evolution.

Only two genes (*rpsU* and *htrA*) had a secondary TSS in both species whereas 8 and 22 secondary TSS were detected only in GAS or in GBS respectively (Table 2*)*. The sequences of the remaining 30 promoters were aligned on the 5′ regions of the orthologous genes. An alignment was obtained for 14 of them but a − 10 sequence could be predicted for only 6 genes.

Fourteen internal TSS were detected in orthologous genes of the two species, while 31 and 91 genes showed an internal TSS only in *S. pyogenes* or *S. agalactiae*, respectively (Table 2). Nine out of the 14 TSS were conserved and located in the last 250 nt of the CDS for genes. These promoters might have a conserved function in directing alternative transcription inside operons. Two TSS were present at an identical position in the 5′ portion of the CDS coding for the dTDP-D-glucose dehydratase RfbB and of the nucleoside transporter NupC. These TSS could initiate either a ncRNA or a transcript coding for a shorter form of the protein. The last 3 internal TSS in sense orientation occurred at different positions relative to the CDS and correspond to different promoters. Antisense transcription originating from an internal TSS or a proximal TSS in intergenic region was poorly conserved, affecting only two orthologous genes in both species (*recU* and *SP119_1603* encoding a potential mechanosensitive ion channel protein).

### Comparison of *S. pyogenes* and *S. agalactiae* 5′ UTR reveals novel potential regulatory sequences

**Fig. 6:**
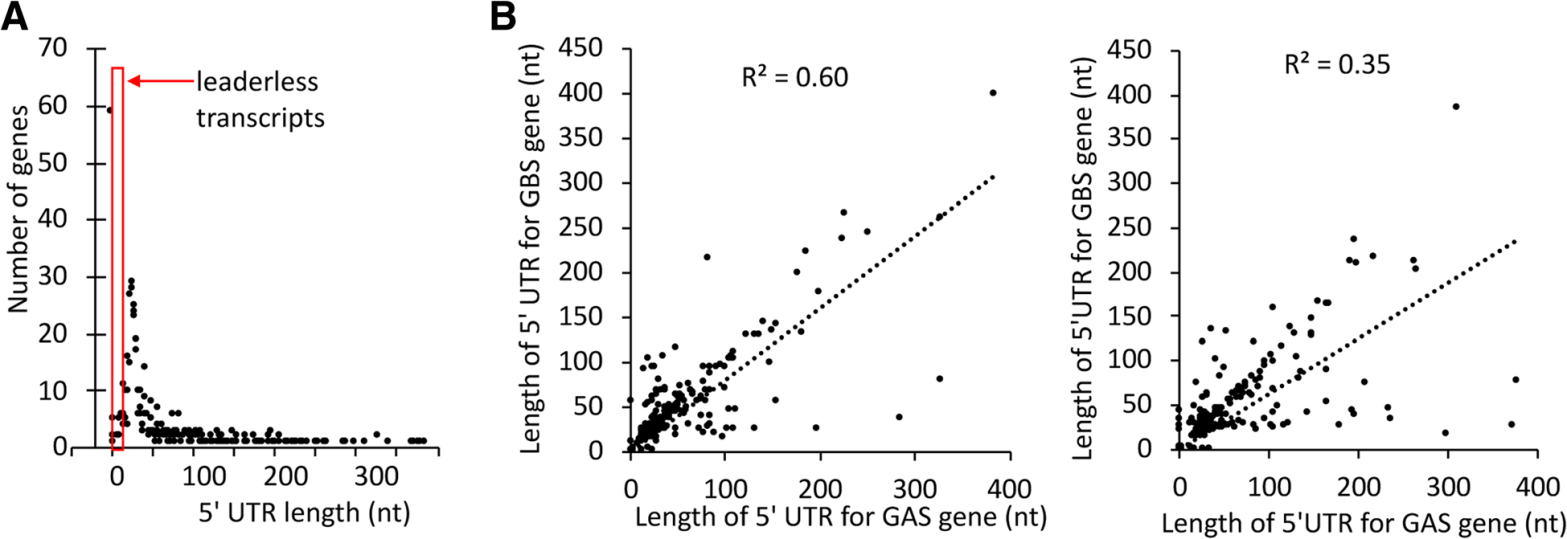
Conservation of 5′ UTR lengths in *S. pyogenes* and *S. agalactiae* genes. **a** Length of 5′ UTR in *S. pyogenes* genes; **b** Comparison of the lengths of *S. pyogenes* and *S. agalactiae* transcript 5′ UTRs of orthologous genes when the synteny with upstream gene was maintained (left panel) or disrupted by genome reorganization (right panel). Dotted lines represent the linear regression curves. The coefficients of determination R^2^ are given

5′ UTR are important determinant of transcript stability and translation efficiency. Analysis of the distance between TSSs and translation initiating codons showed that 337 (43%) of the coding transcriptional units in *S. pyogenes* strain S119 have 5′ UTRs 15- to 35- nt long (Fig. 6a). 8.6% of the mRNAs (*n* = 67) were leaderless, a proportion similar to that described in *S. agalactiae* (9%) and *S. pneumoniae* (9%) [10, 12].

Twenty-six percent of the TU (*n* = 195) had 5′ UTR longer than 60 nt that could potentially be structured and involved in post-transcriptional regulations and three transcripts have 5′ UTR longer than 400 nt. We analyzed the conservation of 5′ UTR lengths between genes shared by *S. pyogenes* and *S. agalactiae* as a function of promoter sequence conservation and rearrangement of the intergenic region. We found a better correlation between the lengths of 5′ UTR in *S. pyogenes* and *S. agalactiae* when the promoter was conserved (coefficient of determination of 0.67 versus 0.4) or when the upstream gene remained unchanged (coefficient of determination of 0.63 versus 0.35) (Fig. 6b). This shows that selective pressure on promoter sequence conservation and the absence of genomic reorganization affecting the intergenic region are major determinants of the conservation of 5′ UTR lengths.

On the other hand, 85 pairs of orthologous genes have 5′ UTR longer than 60 nt in both species, among which 48 had 5′ UTR lengths that vary by less than 10% (see Additional file 11: Table S8). We reasoned that a size conservation of long 5′ UTR might reveal evolutionary constraints on 5′ UTR structure associated with new regulatory sequences. Twenty one of these potential regulatory structures were previously annotated as 5′ cis-regulatory sequences in *S. agalactiae* or *S. pyogenes* or had similarity to identified cis-regulatory sequence families in the Rfam database [42]. Among the remaining 27 sequences, six were previously identified as participating in the autoregulation of ribosomal protein synthesis [43–45] and in *prfB* translational frameshifting [46, 47] although not annotated in Rfam database. Interestingly the 5′ UTR sequence of the *rplk-rplA* operon encoding the L11 and L1 ribosomal proteins was highly conserved among streptococci but its involvement in a regulatory function was only reported in *E. coli* [48]. Alignment and folding prediction by using LocARNA [49] showed that despite the evolutionary distance, this sequence folds similarly in *E. coli* and in streptococci (See Additional file 12: Figure S4), suggesting a conserved mechanism of feedback translational regulation of the operon by the L1 protein. The 5′ UTR of *rpmH* encoding the L34 protein also displayed a strong conservation of sequence and structure among streptococci (See Additional file 13: Figure S5) suggesting a regulatory function to be characterized. The 5′ UTR of the genes encoding the translation elongation factor 1A (See Additional file 14: Figure S6), the cell division protein FtsA (See Additional file 15: Figure S7) and the glyceraldehyde-3-P deshydrogenase (See Additional file 16: Figure S8) show conserved sequence and/or structure with compensatory mutations among diverse streptococci suggesting that these 5′ UTR regulate translation and/or transcript stability of the corresponding genes.

## Discussion

Recent phylopathogenomics studies have highlighted the role of regulatory mutations in driving selection of new bacterial clones with different virulence properties. Although *S. pyogenes* is a major human pathogen and as such has been widely studied, a mapping of its primary transcriptome at the whole-genome scale is still lacking. To fill this gap, we have used a dRNA-seq approch in combination with directional RNA-sequencing to characterize the transcriptome architecture of a M1 clinical isolate of *S. pyogenes* at the single-nucleotide resolution.

In total, we determined 892 TSS, 89% of them mapping in the core genome of the species and shared by other M types. This allowed the characterization of promoter sequences and 5’UTR that are key determinants of gene regulation. We also mapped 643 transcript ends and identified 117 putative regulatory RNAs.

When compared with the 39 TSS previously determined by other methods, 30 TSS we have determined matched with a tolerance of three nt (See Additional file 17: Table S9). The other 9 TSS were either not mapped in our experiment or mapped at a different position. Some of the missing TSS might correspond to genes expressed at low levels under our growth conditions or to serotype-specific differences in gene expression. Alternatively, some of the previously determined 5′ ends, associated with poor promoter sequences, might have been generated through RNase processing such as recently shown for one of the *spe*B transcript 5′ ends [40]. Indeed we also observed a transcript 5′ end at this position but with a TAP+/TAP- ratio close to 1. Compared to previous TSS characterizations our study provides a substantial amount of new information that can be used to identify potential regulatory mutations in M1 type strains as well as in strains of other M types. An annotated sequence containing this information is available to the community under Genbank accession number LR031521.1.

Hundreds of ncRNAs have been previously described by using bioinformatics, microarrays and RNA-seq [13–20], however with limited overlaps between studies. Among the 117 putative regulatory RNAs we identified, sixty nine confirmed ncRNAs detected in these previous studies, also providing information on their 5′ end that was most often lacking. In particular in our screen we retrieved ncRNAs that have been well characterized *in S. pyogenes* or in other species, such as the FasX RNA, Pel RNA, tracrRNA, 4.5S RNA, tmRNA, 6S RNA, csRNAs and the RNA component of RNase P. The only exception was the RivX RNA, however its absence has also previously been noted in the transcriptome of another M1 strain [14]. While the discovery of ncRNAs classified as intergenic or generated from 5’UTR was apparently nearly saturated by previous screens, at least under current laboratory growth conditions, a different situation comes from the characterization of antisense transcripts, among which 36 were novel, including seven lasRNAs. Although some of these ncRNAs were associated with a low coverage and might have occurred through spurious transcription initiations, the high levels of expression of others are in favor of specific regulatory functions.

The transcriptome architecture of *S. pyogenes* was further compared with that of *S. agalactiae,* a distantly related Streptococcus of the pyogenic group. Although remnants of synteny were observed between both streptococci, the two genomes have largely been reshuffled during evolution. This creates an interesting situation where the fate of promoters and 5′ UTR could be compared between pairs of orthologous genes depending on the conservation or not of the synteny with the upstream gene.

This comparative study revealed both conserved and species-specific features. Interestingly, we observed that the proportion of primary TSS relative to the total number of CDS was similar in *S. pyogenes* and in *S. agalactiae* with respectively 40.8 and 42% of coding genes preceded by a primary TSS respectively (Table 2). Furthermore, this proportion was also close to that recently described in *S. pneumoniae* (40.4%; 828 pTSS for 2016 genes/pseudogenes) [12], and therefore could be a conserved property in streptococci. This global conservation was also reflected in the relative conservation of operon architecture between *S. agalactiae* and *S. pyogenes*. Nevertheless, we showed that both the promoter sequence and the 5′ UTR length were more variable when gene synteny was not maintained indicating that genome reshuffling might favor the evolution of new regulatory sequences. While this idea is relatively intuitive, to our knowledge it was not previously tested. In this context, conservation of some regulatory features such as cis-regulatory RNAs or long 5′ UTR may be considered as a sign of an evolutionary pressure indicating a general functional importance.

Fifty four transcripts were classified as leaderless both in *S. pyogenes* and *S. agalactiae* (81% of *S. pyogenes* leaderless transcripts). Furthermore 39 were also classified as leaderless in *S. pneumoniae* [12] revealing a strong selective pressure on the maintenance of transcripts that begin at or very close to the translation initiation codon among distantly related streptococci. Translation of leaderless mRNAs likely occurs through the direct interaction of a 5′-terminal AUG with a pre-formed 70S ribosome [50] and critically depends on the ratio of the initiation factors 2 and 3 [51]. In *E. coli*, an increased translation of leaderless mRNAs was described depending on environmental conditions such as low temperature [52] or stress conditions inducing the release of the anti-Shine-Dalgarno sequence in 16S rRNA by the MazF toxin [53]. Therefore, conservation of leaderless transcripts between *S. pyogenes*, *S. agalactiae* and *S. pneumoniae* might reflect common regulatory mechanisms among the three streptococci.

In contrast to primary promoters, only a limited number of secondary promoters were conserved between the two streptococci; furthermore, only 14 secondary TSS were detected in *S. pyogenes* versus 37 in *S. agalactiae*. In these two species transcription is essentially performed by the RNA polymerase associated with the housekeeping sigma factor and does not use alternative sigma factors. Instead the response to external stimuli is mainly controlled by two-component systems, which may explain the small number of secondary promoters. For 14 of these secondary promoters found in only one species, a sequence alignment could be obtained in the other species but was associated with a likely inactivation of the − 10 sequence in 8 of them. This suggested that although some of the associated TSS may have been missed in one of the species, differences in secondary promoters more likely reflected species-specific evolutions. For instance the larger number of secondary promoters in *S. agalactiae* might be related to its capacity to adapt to a larger number of different hosts.

Promoters lying inside CDS, on the same or opposite strands, were also generally badly conserved among *S. pyogenes* and *S. agalactiae*. Such a difference in the conservation of primary promoters versus internal or antisense promoters was previously reported in a more extended comparative transcriptome analysis across the *Shewanella* genus [54].

Interestingly, the majority of the virulence genes characterized in *S. pyogenes* have no ortholog in *S. agalactiae*. Furthermore some of them, linked to the increased pathogenicity of some M clones, are specific to a small number of lineages. As such, they have only recently been integrated into regulatory networks, showing the strong plasticity of these networks. The CovRS two-component system is a master regulator of virulence, influencing, directly or indirectly, the expression of 10–15% of *S. pyogenes* genome in response to environmental stimuli. In particular the CovS kinase was identified as a major sensor of extracellular Mg^2+^ [26]. ncRNAs might also been involved in these regulatory networks as we found that expression of nine of them was modified under conditions of high Mg^2+^ concentration. Interestingly, these ncRNA include the sag/pel sRNA and two sRNAs controlled by the CiaRH two-component system, csRNA15 and csRNA25 [14]. The relationships between the response to extracellular Mg^2+^ and the CiaRH regulation remains to be explored. In addition, the expression of an antisense ncRNA possibly involved in the regulation of the LacD.1 aldolase, a metabolic sensor regulating SpeB expression [37] was also affected by the Mg^2+^ concentration. SpeB, a major virulence factor in *S. pyogenes*, was previously found to display a surprisingly complex regulation, being controlled at transcriptional, post-transcriptional and post-translational levels. Some of our results, such as the characterization of *speB* and *ropB* transcripts or the description of the antisense transcription on the *lacABCD* operon may help to further decipher these complex regulations.

## Methods

### Bacterial strains and growth conditions

RNA-seq and dRNA-seq experiments were conducted with the M1 type *S. pyogenes* strain S119, an invasive strain isolated from human blood in 2008. Bacteria were cultured to late-exponential phase (OD_600_ = 0.8) in Todd-Hewitt broth with yeast extract (THY) supplemented or not with 15 mM MgCl_2_. For each condition triplicate cultures were obtained.

### Genome sequencing and annotation

S119 complete genome sequencing was carried out by using the Illumina technology, with read length of 51 nt and a more than 200 fold-coverage. De novo assembly was performed by using the SPAdes (SPAdes/3.1.0) software. Genomic sequences of M1 strains SF370 (NC_002737.2) and MGAS5005 (NC_007297.2) were used to reorder contigs by using Mauve [55]. Remaining ambiguous junctions, specially at prophage extremities, were amplified by PCR and sequenced by Sanger sequencing. The sequence was annotated by comparison with the genome sequence annotations of strains SF370 (NC_002737.2) and MGAS5005 (NC_007297.2).

### RNA extraction and rRNA depletion

Total RNA was prepared as previously described [56]. Residual DNA was removed with TURBO DNAse (Ambion). RNA integrity was verified with the Agilent Bioanalyzer 2100. Only RNA preparations with RNA Integrity Numbers greater than 9 were kept for analyses. mRNA enrichment was performed with the MICROBExpress Kit (Ambion). Depletion of 16S and 23S ribosomal RNAs was confirmed with the Agilent Bioanalyzer 2100.

### dRNA-seq and RNA-seq experiments

For dRNA-seq a pool of RNA was obtained by mixing three RNA extractions from S119 cultured in THY medium and three RNA extractions from S119 cultured in THY medium supplemented by MgCl_2_ 15 mM. Strand-specific RNA-seq was conducted on the six RNAs taken individually. Library preparations were constructed as previously described [10, 25]. Sequencing was performed on the Illumina HiSeq 2000 using 50 sequencing cycles.

### dRNA-seq and RNA-seq analyses

Sequencing reads generated from dRNA-seq and RNA-seq libraries were trimmed for adapter sequences with Cutadapt [57] and reads shorter than 18 nucleotides (dRNA-seq) or 20 nucleotides (RNA-seq) were discarded. Mapping was performed on *S119* genome sequence by using Bowtie (version 0.12.7) [58] and reads that mapped at more than four different positions on the genome were discarded, i. e. reads corresponding to rRNA. For dRNA-seq, a statistical assignment of TSS positions was performed by EdgeR (version 3.2.4) [59] as previously described [10]. *p*-values after multiple testing adjustment procedure [60] were calculated leading to the assignment of 528 TSS with FDR ≤ 0.1 (TSS designated as “A” in Sup. Table 2). As this procedure was previously found to be generally too stringent, eliminating many true TSSs, 271 additional TSS were predicted by considering positions where the raw p-values were less than 0.05 and that could be confirmed by visual inspection with the IGV genome browser [61] based on dRNA-seq and RNA-seq data (TSS designated as “B” in Sup. Table 2). Finally 94 TSSs were only determined based on RNA-seq data when TAP+/TAP- difference was not significant but a putative promoter was predicted and the 5′ end does not result of the cleavage of a longer precursor (designated as “C” in Sup. Table 2). RNA-seq data were analyzed as described [10] using Rsamtools (version 1.26.2), GenomicAlignments (version 1.10.1), GenomicFeatures (version 1.26.4) in R 3.3.1. For differential expression analysis, normalization and statistical analyses were performed by using DESeq2 (version 1.14.1). Only genes with Fold Change ≥2 and p-values ≤0.05 after multiple testing adjustment procedure [60] were considered as differentially expressed.

### Determination of operon structure and mapping of transcript 3′ ends

To map transcript 3′ ends, the coverage per nucleotide was determined along both strands of the genome sequence by using the SAMtools (version 0.1.12a) and variations in coverage were calculated with a custom R script as previously described [10]. The custom R script is provided as Additional file 18. The list of the transcript ends was compared with the positions of potential terminators as described by de Hoon et al. [31]. Supplementary terminators were searched with ARNold [32] (http://rna.igmors.u-psud.fr/toolbox/arnold/) and TransTermHP (http://transterm.cbcb.umd.edu). Two consecutive genes in the same orientation were considered as belonging to different transcription units (TU) if transcription of the first gene ends with a 100% efficient termination site or if a primary TSS was detected upstream of the second gene. Accordingly, TU were classified into one of three categories: 1) monocistronic TU; 2) simple operons, composed of several genes preceded by a primary TSS and separated from the next TU by a 100% efficient termination site and/or a primary TSS; 3) composite operons preceded by a primary TSS and characterized by the presence of at least one internal TSS and/or one inefficient terminator leading to variations in gene expression levels along the operon.

### Comparisons with *S. agalactiae* transcriptome

To calculate the phylogenetic distance between *S. pyogenes* and *S. agalactiae*, we used the 136 genes defining the core genome of the *Streptococcus* genus as identified in [62]. The sequences of the corresponding CDS in the *S. agalactiae* strain NEM316 (NC_004368.1) and the *S. pyogenes* strain S119 were concatenated and aligned using clustalW implemented in Mega version 7 [63]. The pairwise distances were computed to estimate the number of amino acid substitutions per site. Analyses were conducted using the Poisson correction model. All positions containing gaps and missing data were eliminated, leading to a final dataset of 32,649 positions. Genes conserved between both species were defined by reciprocal best hits using BlastP alignments (version ncbi-blast-2.5.0). Only pairs of shared genes with sequence identity of no less than 40% were kept. To quantify the conservation of promoter sequences the 50 nt upstream of each TSS were extracted and aligned against sequences from the other species: i) for primary and secondary TSS: a 550 nt long sequence encompassing the 500 nt preceding the translation initiation codon and the 50 first nt of the CDS of orthologous CDS ii) for internal TSS: the nucleotide sequence of the homologous CDS; and iii) for antisense TSS: the complementary sequence of the CDS plus the 250 nt upstream and downstream sequences. Alignments were performed with BlastN adapted for short sequence (word-size of 7) and a E-value threshold of 0.001. Based on this alignment, the position of the TSS was calculated and compared with the position determined experimentally. Promoters were considered as conserved when both positions coincided in a window of three nucleotides.

## Additional files

Additional file 1: **Table S1**. Number of reads mapped in the dRNA-Seq and RNA-seq experiments. (PDF 44 kb)

Additional file 2: **Table S2**. TSS detected in the study. (XLSX 138 kb)

Additional file 3: **Figure S1**. Nucleotide usage in reiterative transcription identified in 113 TSSs. Pie chart of the occurrence of pseudo-templated nucleotides at the transcription initiation sites in *S. pyogenes*. (PDF 282 kb)

Additional file 4: **Table S3**. Predicted positions of gene TSS in representative strains of other M types. (XLSX 148 kb)

Additional file 5: **Table S4**. Identification of transcript 3′ ends and presence of a predicted rho-independent terminator. (XLSX 62 kb)

Additional file 6: **Table S5**. Operon map predicted in strain S119. (XLSX 61 kb)

Additional file 7: **Figure S2**. Processing of *pnp* 5′ UTR. A) dRNA-seq and RNA-seq reads aligning to *pnp* gene visualized by IGV. dRNA-seq and RNA-seq experiments detect two transcript-5′-ends: a tri-P 5′-end resulting from a TSS upstream a 22 nt-long sRNA and a mono-P 5′-end upstream the mature RNA and present at the same level among reads generated with and without TAP treatment and predicted to result from an endonucleolytic processing of the primary *pnp* mRNA. The strand-specific RNA-seq identifies two transcripts: one 22 nt-long sRNA beginning at the TSS and the *pnp* transcript beginning at the processing site. B. Sequence of the *pnp* primary transcript 5′ UTR showing the 22 nt-sRNA part in red and the beginning of the 6.6 kb mature transcript corresponding to *pnp-S119_1643–1642–1641-1640-1639-1638* operon in green. C. Folding of the *pnp* primary transcript 5′ UTR showing the position of the 3′ end of the sRNA and of the 5′ end of the *pnp* mature transcript staggered two base-pairs apart in a long stem loop. (PDF 805 kb)

Additional file 8: **Table S6**. ncRNAs detected in the study. (XLSX 26 kb)

Additional file 9: **Figure S3**. Characterization of a new family of sRNAs potentially regulating expression of prophage integrases. A. Position of the four ncRNAS antisense to the 5′ UTR of the integrase genes (*Int*) of the four prophages S119-P1, −P2, −P3, −P4 of strain S119. B. Sequence alignment of the ncRNAS. (PDF 375 kb)

Additional file 10: **Table S7**. Genes regulated in response to the presence of high Mg2+ concentrations (XLSX 21 kb)

Additional file 11: **Table S8**. Characteristics of long 5′ UTRs conserved between *S. pyogenes* and *S. agalactiae. (XLSX 15 kb)*

Additional file 12: **Figure S4**. DNA sequence alignment and structure prediction of the 5′ UTR of the *rplK-rplA* operon encoding the ribosomal proteins L11 and L1. The DNA sequences of the 5′ UTR in ten streptococci and in *E.coli* were extracted from Genbank. The 5′ UTR sequence was predicted by checking for the presence of a potential − 10 box 7–9 nt upstream of the first nucleotide. Alignment and folding prediction were performed by using LocARNA (http://rna.informatik.uni-freiburg.de/LocARNA). Compatible base pairs are colored, where the hue shows the number of different types C-G, G-C, A-U, U-A, G-U or U-G of compatible base pairs in the corresponding columns. The saturation decreases with the number of incompatible base pairs.Accession numbers: *S. agalactiae:* NC_004368.1; *S. gallolyticus*: CP013688.1; *S. infantarius*: CP013689.1; *S. mitis*: CP014326.1; *S. pneumoniae*: CP016633.2; *S. uberis*: NC_012004.1; *S. parauberis*: CP025420.1; *S. equi*: LS483325.1; *S. iniae*: CP024843.1; *E. coli*: NC_000913.3. (PDF 566 kb)

Additional file 13: **Figure S5**. DNA sequence alignment and structure prediction of *rpmH* 5′ UTR. The DNA sequences of r*pmH* 5′ UTR in ten streptococci were extracted from NCBI sequence database. The 5′ UTR sequence was predicted by checking for the presence of a potential − 10 box 7–9 nt upstream of the first nucleotide. Alignment and folding prediction were performed by using LocARNA (http://rna.informatik.uni-freiburg.de/LocARNA). Compatible base pairs are colored, where the hue shows the number of different types C-G, G-C, A-U, U-A, G-U or U-G of compatible base pairs in the corresponding columns. The saturation decreases with the number of incompatible base pairs. Accession numbers: *S. agalactiae:* NC_004368.1; *S. gallolyticus*: CP013688.1; S. mutans: NC_004350.2; *S. pneumoniae*: CP016633.2; *S. salivarius*: CP014144.1; *S. suis*: NC_012926.1; *S. thermophilus*: CP016877; *S. uberis*: NC_012004.1; *S. equi*: LS483328.1. (PDF 502 kb)

Additional file 14: **Figure S6**. DNA sequence alignment and structure prediction of the 5′ UTR of *tuf* encoding the EF-TU factor. The DNA sequences of r*pmH* 5′ UTR in ten streptococci were extracted from NCBI sequence database. The 5′ UTR sequence was predicted by checking for the presence of a potential − 10 box 7–9 nt upstream of the first nucleotide. Alignment and folding prediction were performed by using LocARNA (http://rna.informatik.uni-freiburg.de/LocARNA). Compatible base pairs are colored, where the hue shows the number of different types C-G, G-C, A-U, U-A, G-U or U-G of compatible base pairs in the corresponding columns. The saturation decreases with the number of incompatible base pairs. Accession numbers: *S. agalactiae:* NC_004368.1; *S. dysgalactiae*: CP002215.1; *S. gallolyticus*: CP013688.1; *S. mitis*: CP014326.1; *S. pneumoniae*: CP016633.2; *S. salivarius*: CP014144.1; *S. suis*: NC_012926.1; *S. thermophilus*: CP016877; *S. equi*: LS483325.1. (PDF 495 kb)

Additional file 15: **Figure S7**. DNA sequence alignment and structure prediction of the 5′ UTR of *ftsA* . The DNA sequences of *ftsA* 5′ UTR in six streptococci were extracted from NCBI sequence database. The 5′ UTR sequence was predicted by checking for the presence of a potential − 10 box 7–9 nt upstream of the first nucleotide. Alignment and folding prediction were performed by using LocARNA (http://rna.informatik.uni-freiburg.de/LocARNA). Compatible base pairs are colored, where the hue shows the number of different types C-G, G-C, A-U, U-A, G-U or U-G of compatible base pairs in the corresponding columns. The saturation decreases with the number of incompatible base pairs. Accession numbers: *S. agalactiae:* NC_004368.1; *S. gallolyticus*: NC_017576.1; *S. mutans*: NC_004350.2; *S. thermophilus*: CP016877; *S. uberis*: NC_012004.1. (PDF 580 kb)

Additional file 16: **Figure S8**. DNA sequence alignment and structure prediction of the 5′ UTR of *gapD*. The DNA sequences of *gapD* 5′ UTR of eight streptococci were extracted from NCBI sequence database. The 5′ UTR sequence was predicted by checking for the presence of a potential − 10 box 7–9 nt upstream of the first nucleotide. Alignment and folding prediction were performed by using LocARNA (http://rna.informatik.uni-freiburg.de/LocARNA). Compatible base pairs are colored, where the hue shows the number of different types C-G, G-C, A-U, U-A, G-U or U-G of compatible base pairs in the corresponding columns. The saturation decreases with the number of incompatible base pairs. Accession numbers: *S. agalactiae:* NC_004368.1; *S. dysgalactiae*: CP002215.1; *S. gallolyticus*: NC_017576.1; *S. mutans*: NC_004350.2; *S. pneumoniae*: CP016633.2; *S. salivarius*: CP014144.1; *S. suis*: NC_012926.1; *S. thermophilus*: CP016877; *S. uberis*: NC_012004.1. (PDF 540 kb)

Additional file 17: **Table S9**. Comparison between TSS identified in this study and in previous studies. (XLSX 15 kb)

Additional file 18: R scripts used to determine 3′ ends of transcripts from RNA-seq data. RNA-seq libraries were obtained by using the primer-ligation method as described in Methods. The file provides a copy of the three scripts successively used from the RNA-seq read files to the final table (script1: “coverage_table”; Script2: Table_Annot_script; Script3: Terminator), as well as examples of the Tables used as entry points for running the scripts (Infogen.txt, table soft.txt, table RunInfo.txt, table CDS.txt). (TXT 65 kb)

## Abbreviations

CDS: Coding sequence
Cov: Control of virulence
dRNA-seq: differential RNA-sequencing
FDR: False discovery rate
GAS: Group A *Streptococcus*
GBS: Group B *Streptococcus*
las: Long antisense RNAs
ncRNAs: non-coding RNAs
PBP: Peptidoglycan-binding protein
Pnp: Polyribonucleotide phosphorylase
pTSS: primary TSS
RNA-seq: RNA-sequencing
RopB: Regulator of protease B
RPKM: Reads Per Kilobase Million
*S. agalactiae*: *Streptococcus agalactiae*
*S. pyogenes*: *Streptococcus pyogenes*
SNP: Single-nucleotide polymorphisms
SpeB: Streptococcal pyrogenic exotoxin B
TAP: Tobacco Acid Pyrophosphatase
THY: Todd-Hewitt broth
TSS: Transcription start site
TU: Transcriptional unit
UTR: Untranslated region
