# Supplementary material for: Conserved and specific features of Streptococcus pyogenes and Streptococcus agalactiae transcriptional landscapes
Source: BMC Genomics. 2019 Mar 22;20:236. doi: 10.1186/s12864-019-5613-5 (PMC6431027; doi:10.1186/s12864-019-5613-5)
Supplement: Supplementary file 1 — Table S1. Number of reads mapped in the dRNA-Seq and RNA-seq experiments. (PDF 44 kb) [file 12864_2019_5613_MOESM1_ESM.pdf]

Supplementary Table 1: Number of reads mapped in the dRNA-Seq and RNA-seq experiments

	**TSS mapping**		**Directional RNA-seq libraries**					
	TAP+	TAP-						
Condition	THY and THY, Mg^{++}	THY and THY, Mg^{++}	THY	THY	THY	THY, Mg^{++}	THY, Mg^{++}	THY, Mg^{++}
Number of reads after adapter trimming	23.4 M	21.5 M	33.8 M	30.1 M	31.1 M5	31.1 M	36.1 M	32.1 M
Mapped reads	22.5 M	20.6 M	28.4M	28.4 M	25.5 M	25.7 M	29.9 M	27.4 M
Number of reads mapped to non rRNA regions	5.4 M	2.3 M	18.3 M	11.1 M	17.3 M	17.6 M	19.2 M	17.0 M
