# Supplementary figures and images for: Conserved and specific features of Streptococcus pyogenes and Streptococcus agalactiae transcriptional landscapes
Source: BMC Genomics. 2019 Mar 22;20:236. doi: 10.1186/s12864-019-5613-5 (PMC6431027; doi:10.1186/s12864-019-5613-5)

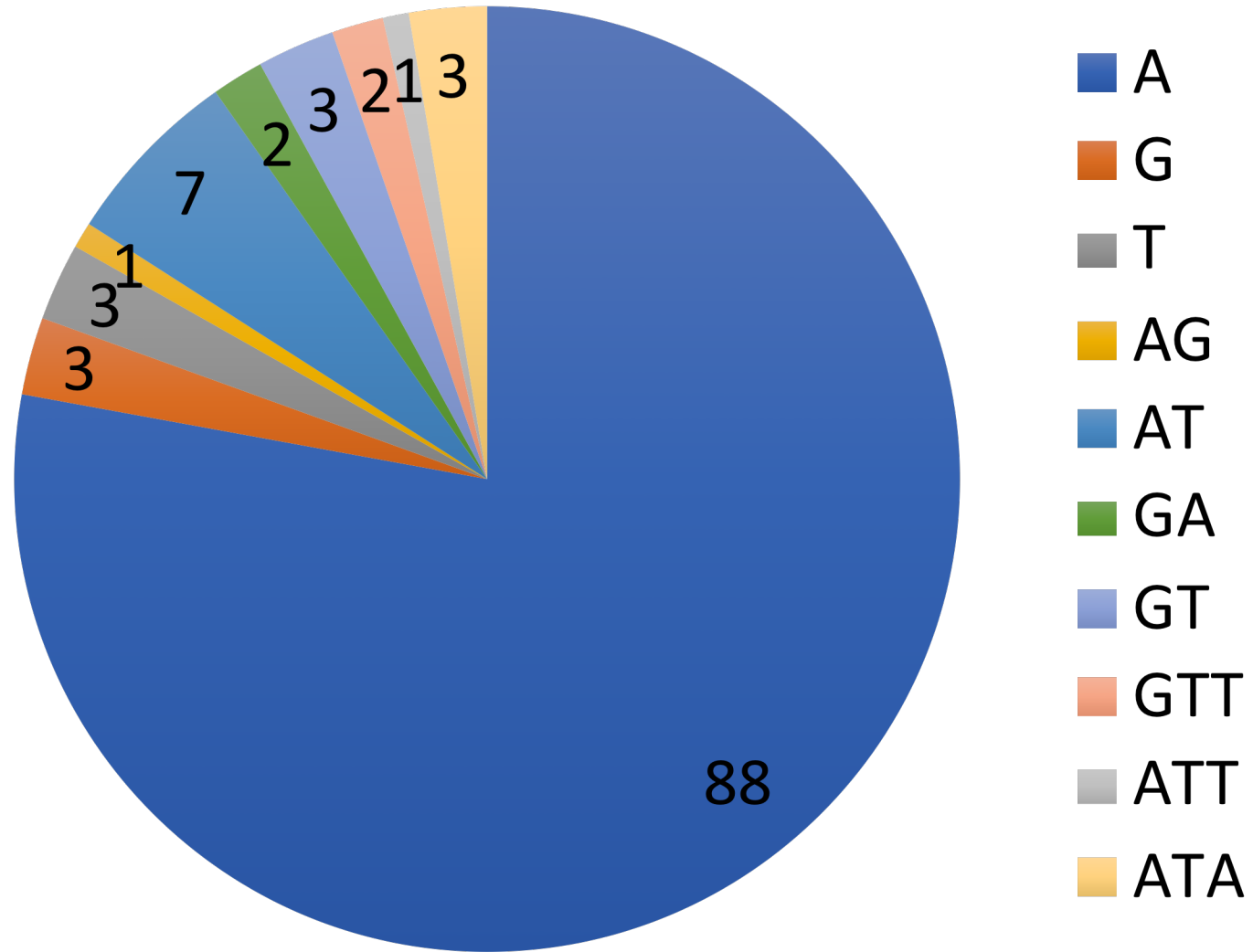

Supplement: Supplementary file 3 — Figure S1. Nucleotide usage in reiterative transcription identified in 113 TSSs. Pie chart of the occurrence of pseudo-templated nucleotides at the transcription initiation sites in S. pyogenes. (PDF 282 kb) [file 12864_2019_5613_MOESM3_ESM.pdf]

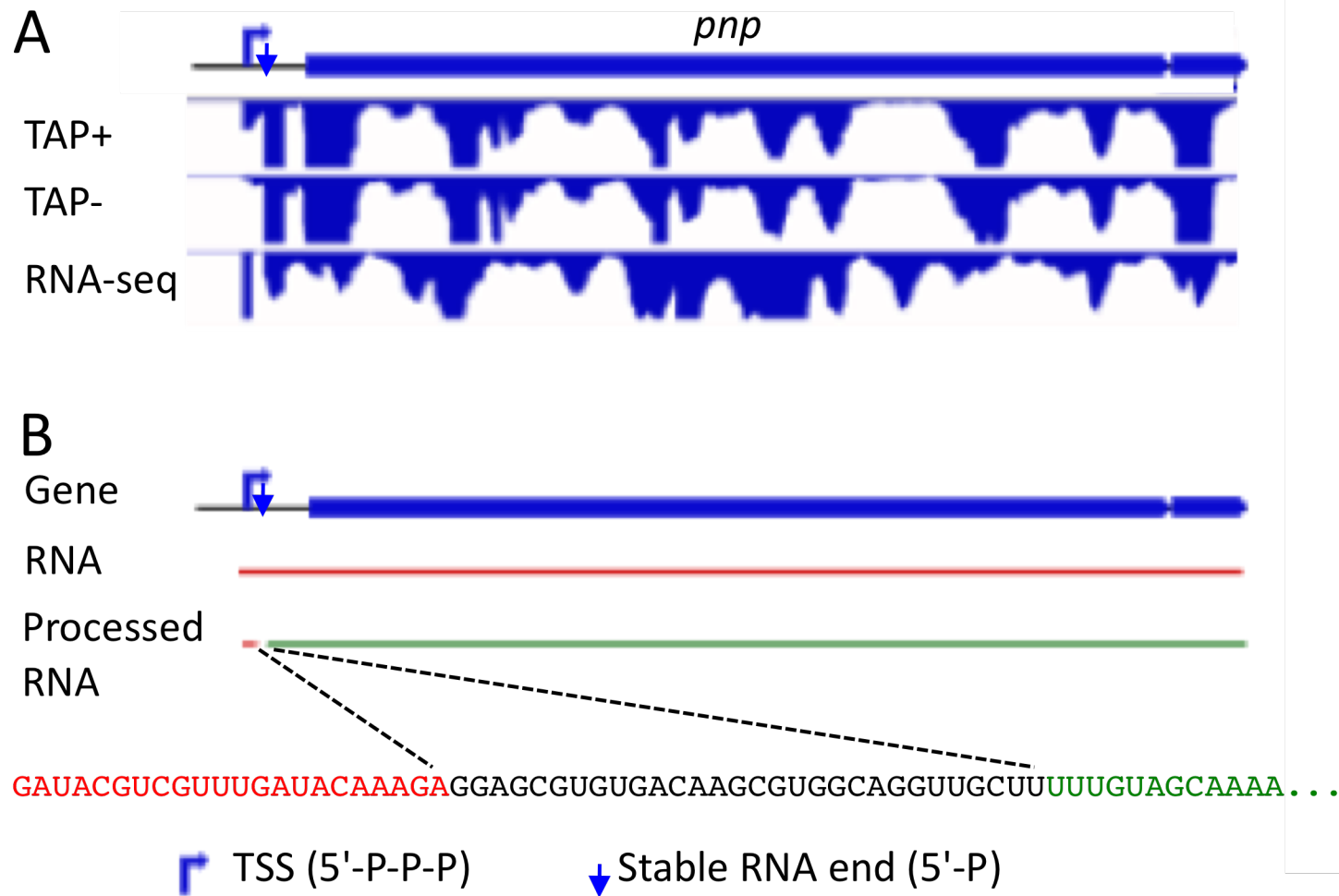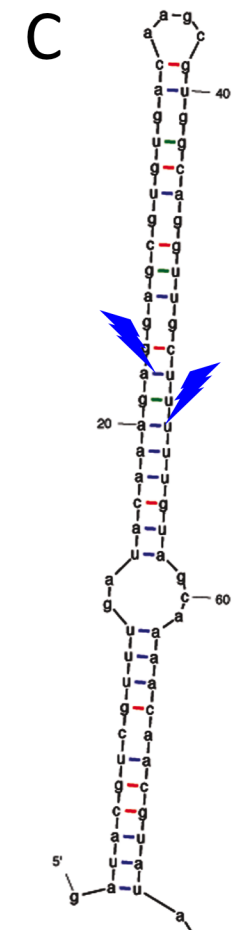

Supplement: Supplementary file 7 — Figure S2. Processing of pnp 5′ UTR. A) dRNA-seq and RNA-seq reads aligning to pnp gene visualized by IGV. dRNA-seq and RNA-seq experiments detect two transcript-5′-ends: a tri-P 5′-end resulting from a TSS upstream a 22 nt-long sRNA and a mono-P 5′-end upstream the mature RNA and present at the same level among reads generated with and without TAP treatment and predicted to result from an endonucleolytic processing of the primary pnp mRNA. The strand-specific RNA-seq identifies two transcripts: one 22 nt-long sRNA beginning at the TSS and the pnp transcript beginning at the processing site. B. Sequence of the pnp primary transcript 5′ UTR showing the 22 nt-sRNA part in red and the beginning of the 6.6 kb mature transcript corresponding to pnp-S119_1643–1642–1641-1640-1639-1638 operon in green. C. Folding of the pnp primary transcript 5′ UTR showing the position of the 3′ end of the sRNA and of the 5′ end of the pnp mature transcript staggered two base-pairs apart in a long stem loop. (PDF 805 kb) [file 12864_2019_5613_MOESM7_ESM.pdf]

A

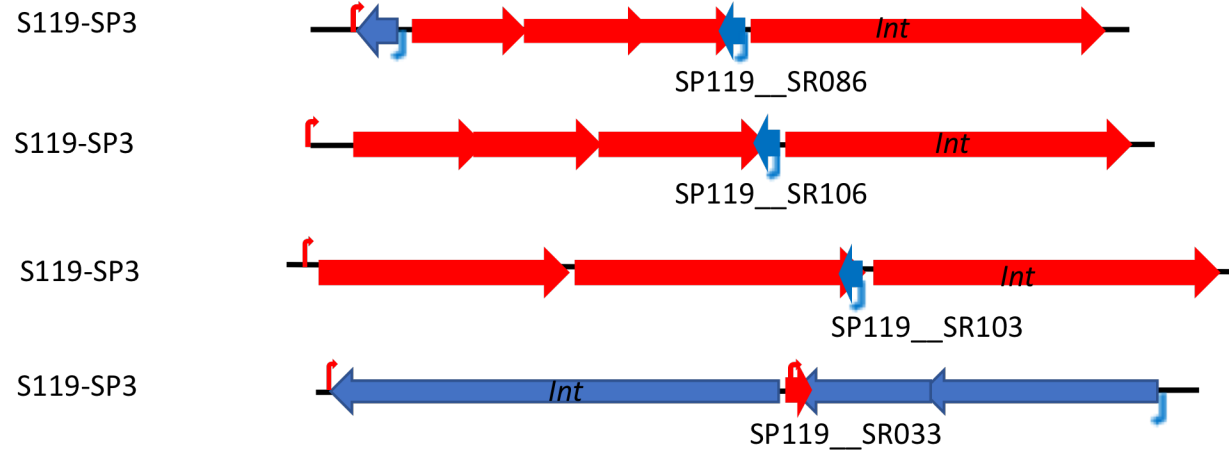

B

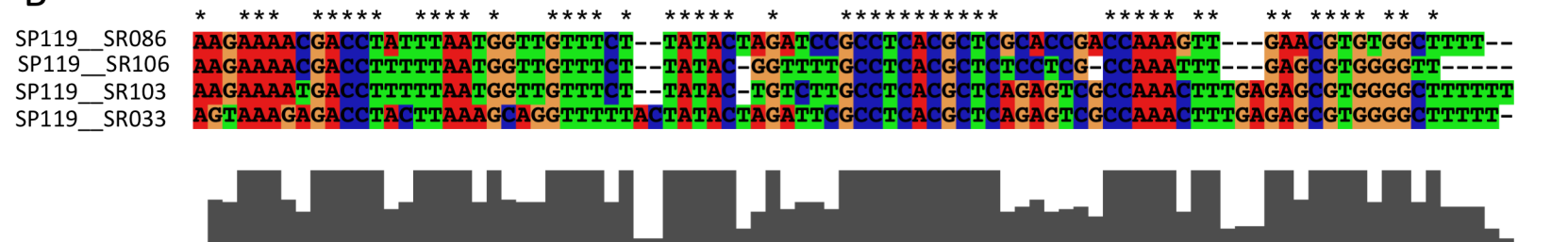

Supplement: Supplementary file 9 — Figure S3. Characterization of a new family of sRNAs potentially regulating expression of prophage integrases. A. Position of the four ncRNAS antisense to the 5′ UTR of the integrase genes (Int) of the four prophages S119-P1, −P2, −P3, −P4 of strain S119. B. Sequence alignment of the ncRNAS. (PDF 375 kb) [file 12864_2019_5613_MOESM9_ESM.pdf]

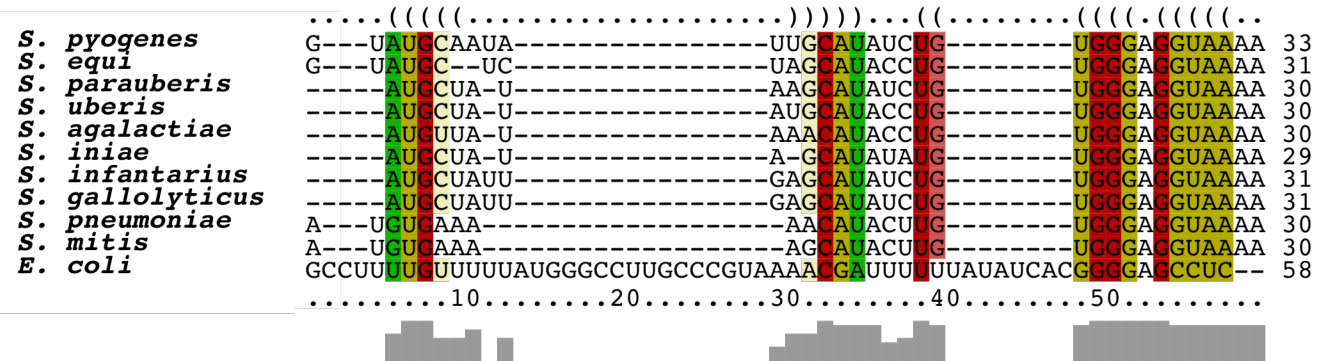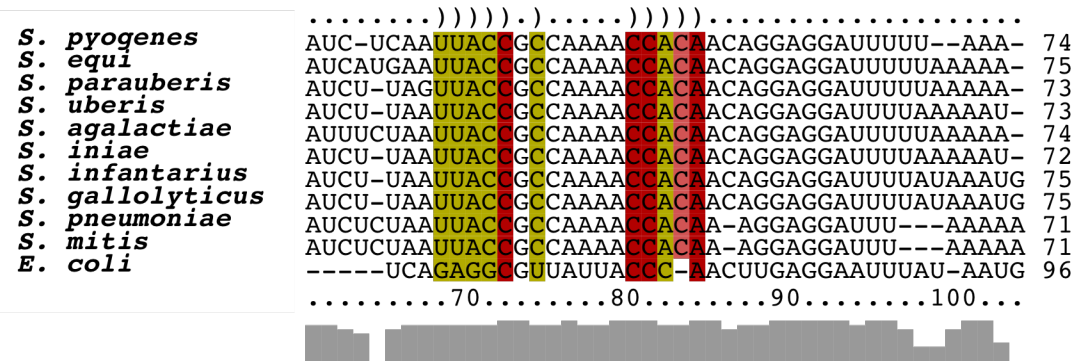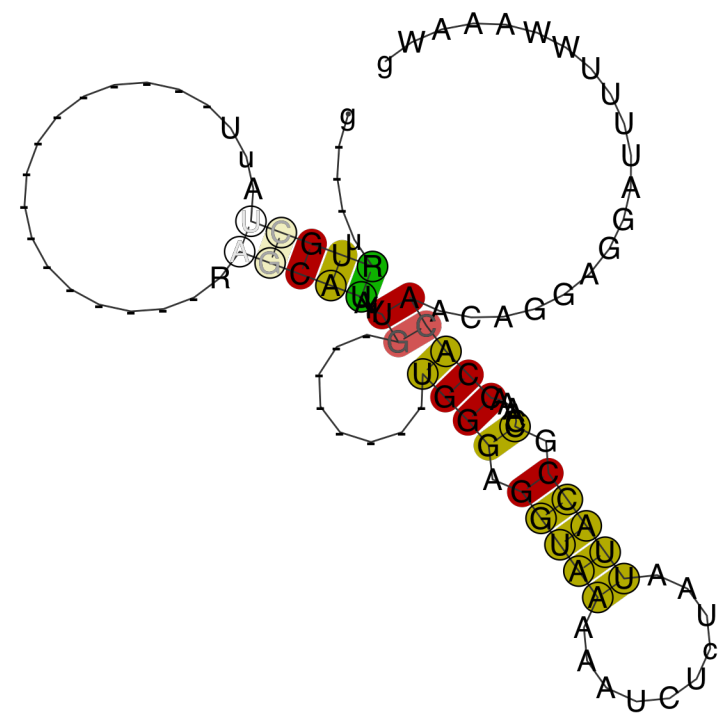

Supplement: Supplementary file 12 — Figure S4. DNA sequence alignment and structure prediction of the 5′ UTR of the rplK-rplA operon encoding the ribosomal proteins L11 and L1. The DNA sequences of the 5′ UTR in ten streptococci and in E.coli were extracted from Genbank. The 5′ UTR sequence was predicted by checking for the presence of a potential − 10 box 7–9 nt upstream of the first nucleotide. Alignment and folding prediction were performed by using LocARNA (http://rna.informatik.uni-freiburg.de/LocARNA). Compatible base pairs are colored, where the hue shows the number of different types C-G, G-C, A-U, U-A, G-U or U-G of compatible base pairs in the corresponding columns. The saturation decreases with the number of incompatible base pairs.Accession numbers: S. agalactiae: NC_004368.1; S. gallolyticus: CP013688.1; S. infantarius: CP013689.1; S. mitis: CP014326.1; S. pneumoniae: CP016633.2; S. uberis: NC_012004.1; S. parauberis: CP025420.1; S. equi: LS483325.1; S. iniae: CP024843.1; E. coli: NC_000913.3. (PDF 566 kb) [file 12864_2019_5613_MOESM12_ESM.pdf]

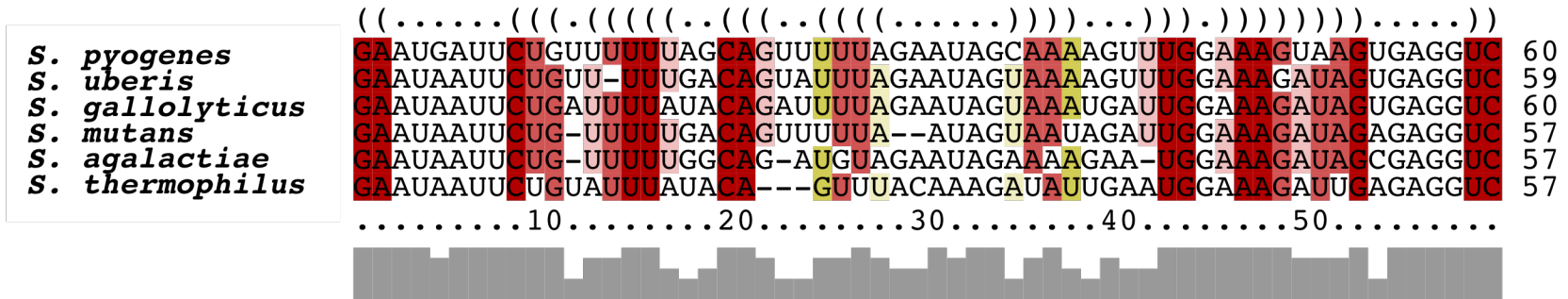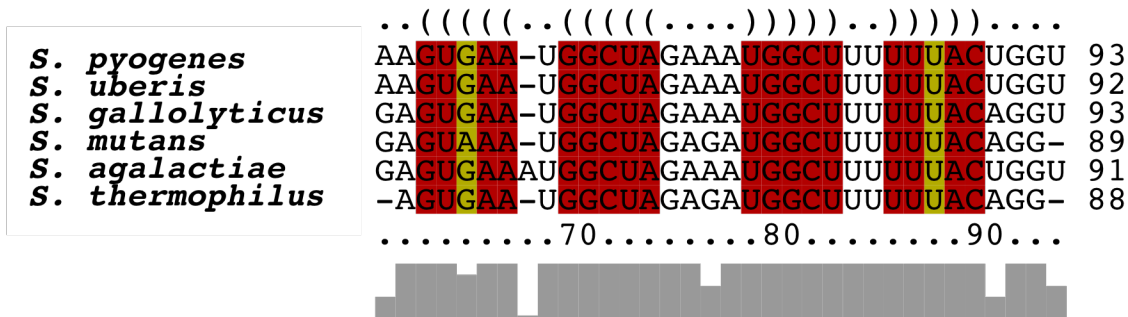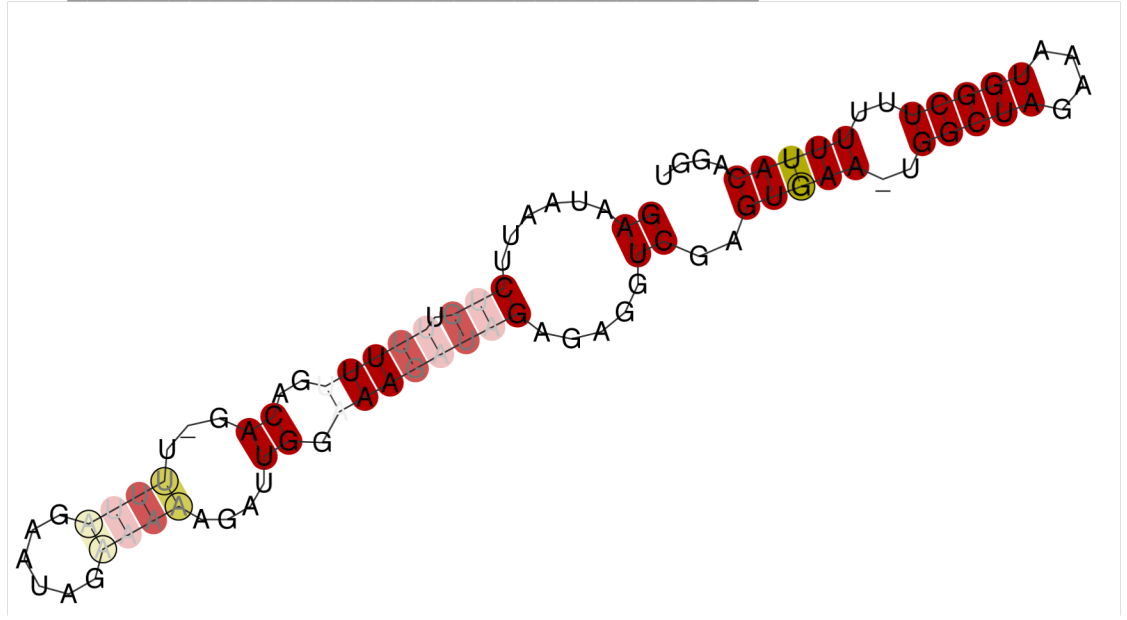

Supplement: Supplementary file 15 — Figure S7. DNA sequence alignment and structure prediction of the 5′ UTR of ftsA . The DNA sequences of ftsA 5′ UTR in six streptococci were extracted from NCBI sequence database. The 5′ UTR sequence was predicted by checking for the presence of a potential − 10 box 7–9 nt upstream of the first nucleotide. Alignment and folding prediction were performed by using LocARNA (http://rna.informatik.uni-freiburg.de/LocARNA). Compatible base pairs are colored, where the hue shows the number of different types C-G, G-C, A-U, U-A, G-U or U-G of compatible base pairs in the corresponding columns. The saturation decreases with the number of incompatible base pairs. Accession numbers: S. agalactiae: NC_004368.1; S. gallolyticus: NC_017576.1; S. mutans: NC_004350.2; S. thermophilus: CP016877; S. uberis: NC_012004.1. (PDF 580 kb) [file 12864_2019_5613_MOESM15_ESM.pdf]
