# Supplementary material for: Conserved and specific features of Streptococcus pyogenes and Streptococcus agalactiae transcriptional landscapes
Source: BMC Genomics. 2019 Mar 22;20:236. doi: 10.1186/s12864-019-5613-5 (PMC6431027; doi:10.1186/s12864-019-5613-5)

```
                ..((((((((((.(((.....))).)))))(((((((((....))))).)))).))))).
S. gallolyticus GUAAAGUUAGACUGUAUUGCUUACCGUCUAUCUAUAAAAUAUAUUUUAUUGGAGGCUUUU 60
S. suis         GUAAAGUUAGACUGUAUUGCCUACUGUCUAUCUAUAAAAUAUAUUUUAUUGGAGGCUUUU 60
S. pyogenes     GUAAAGUUAGACUGUAUGGCCUACCGUCUAUCUAUAAAAUAUAUUUUAUUGGAGGCUUUU 60
S. agalactiae   GUAAAGUUAGACUGUAUUGCCUACCGUCUAUCUAUAAAAUAUAUUUUAUUGGAGGCUUUU 60
S. equi         GUAAAGUUAGACUGUAUUGCCUACCGUCUAUCUAUAAAAUAUAUUUUAUUGGAGGCUUUU 60
S. dysgalactiae GUAAAGUUAGACUGUAUUGCCUACCGUCUAUCUAUAAAAUAUAUUUUAUUGGAGGCUUUU 60
S. thermophilus GUAAAGUUAGACUGUAUUGCCUACUGUCUAUCUAUAAAAUAUAUUUUAUUGGAGGCUUUU 60
S. salivarius   GUAAAGUUAGACUGUAUUGCCUACUGUCUAUCUAUAAAAUAUAUUUUAUUGGAGGCUUUU 60
S. mitis        --AAAGUUAGACUGUAUUGCCUACUGUCUAUCUAUAAAAUAUAUUUUAUUGGAGGCUUUU 58
S. pneumoniae   --AAAGUUAGACUGUAUUGCCUACUGUCUAUCUAUAAAAUAUAUUUUAUUGGAGGCUUUU 58
                .........10........20........30........40........50.........

                .......
S. gallolyticus ACCCAAA 67
S. suis         ACCAAAA 67
S. pyogenes     CCUAAAA 67
S. agalactiae   CCUAAAA 67
S. equi         CCUAAAA 67
S. dysgalactiae CCUAAAA 67
S. thermophilus CCU-AAA 66
S. salivarius   CCU-AAA 66
S. mitis        ACUCAAA 65
S. pneumoniae   ACUCAAA 65
                .......
```

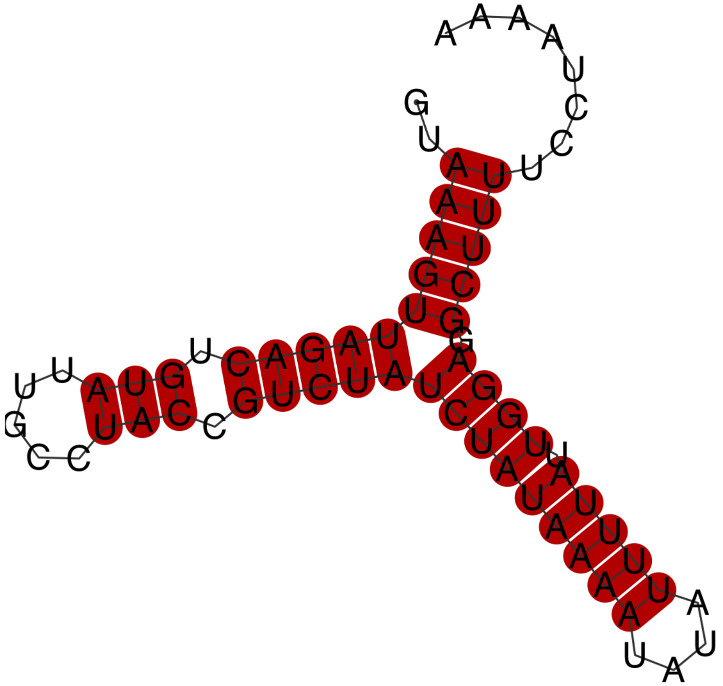

Supplement: Supplementary file 14 — Figure S6. DNA sequence alignment and structure prediction of the 5′ UTR of tuf encoding the EF-TU factor. The DNA sequences of rpmH 5′ UTR in ten streptococci were extracted from NCBI sequence database. The 5′ UTR sequence was predicted by checking for the presence of a potential − 10 box 7–9 nt upstream of the first nucleotide. Alignment and folding prediction were performed by using LocARNA (http://rna.informatik.uni-freiburg.de/LocARNA). Compatible base pairs are colored, where the hue shows the number of different types C-G, G-C, A-U, U-A, G-U or U-G of compatible base pairs in the corresponding columns. The saturation decreases with the number of incompatible base pairs. Accession numbers: S. agalactiae: NC_004368.1; S. dysgalactiae: CP002215.1; S. gallolyticus: CP013688.1; S. mitis: CP014326.1; S. pneumoniae: CP016633.2; S. salivarius: CP014144.1; S. suis: NC_012926.1; S. thermophilus: CP016877; S. equi: LS483325.1. (PDF 495 kb) [file 12864_2019_5613_MOESM14_ESM.pdf]
