# Supplementary material for: Conserved and specific features of Streptococcus pyogenes and Streptococcus agalactiae transcriptional landscapes
Source: BMC Genomics. 2019 Mar 22;20:236. doi: 10.1186/s12864-019-5613-5 (PMC6431027; doi:10.1186/s12864-019-5613-5)
Supplement: Supplementary file 16 — Figure S8. DNA sequence alignment and structure prediction of the 5′ UTR of gapD. The DNA sequences of gapD 5′ UTR of eight streptococci were extracted from NCBI sequence database. The 5′ UTR sequence was predicted by checking for the presence of a potential − 10 box 7–9 nt upstream of the first nucleotide. Alignment and folding prediction were performed by using LocARNA (http://rna.informatik.uni-freiburg.de/LocARNA). Compatible base pairs are colored, where the hue shows the number of different types C-G, G-C, A-U, U-A, G-U or U-G of compatible base pairs in the corresponding columns. The saturation decreases with the number of incompatible base pairs. Accession numbers: S. agalactiae: NC_004368.1; S. dysgalactiae: CP002215.1; S. gallolyticus: NC_017576.1; S. mutans: NC_004350.2; S. pneumoniae: CP016633.2; S. salivarius: CP014144.1; S. suis: NC_012926.1; S. thermophilus: CP016877; S. uberis: NC_012004.1. (PDF 540 kb) [file 12864_2019_5613_MOESM16_ESM.pdf]

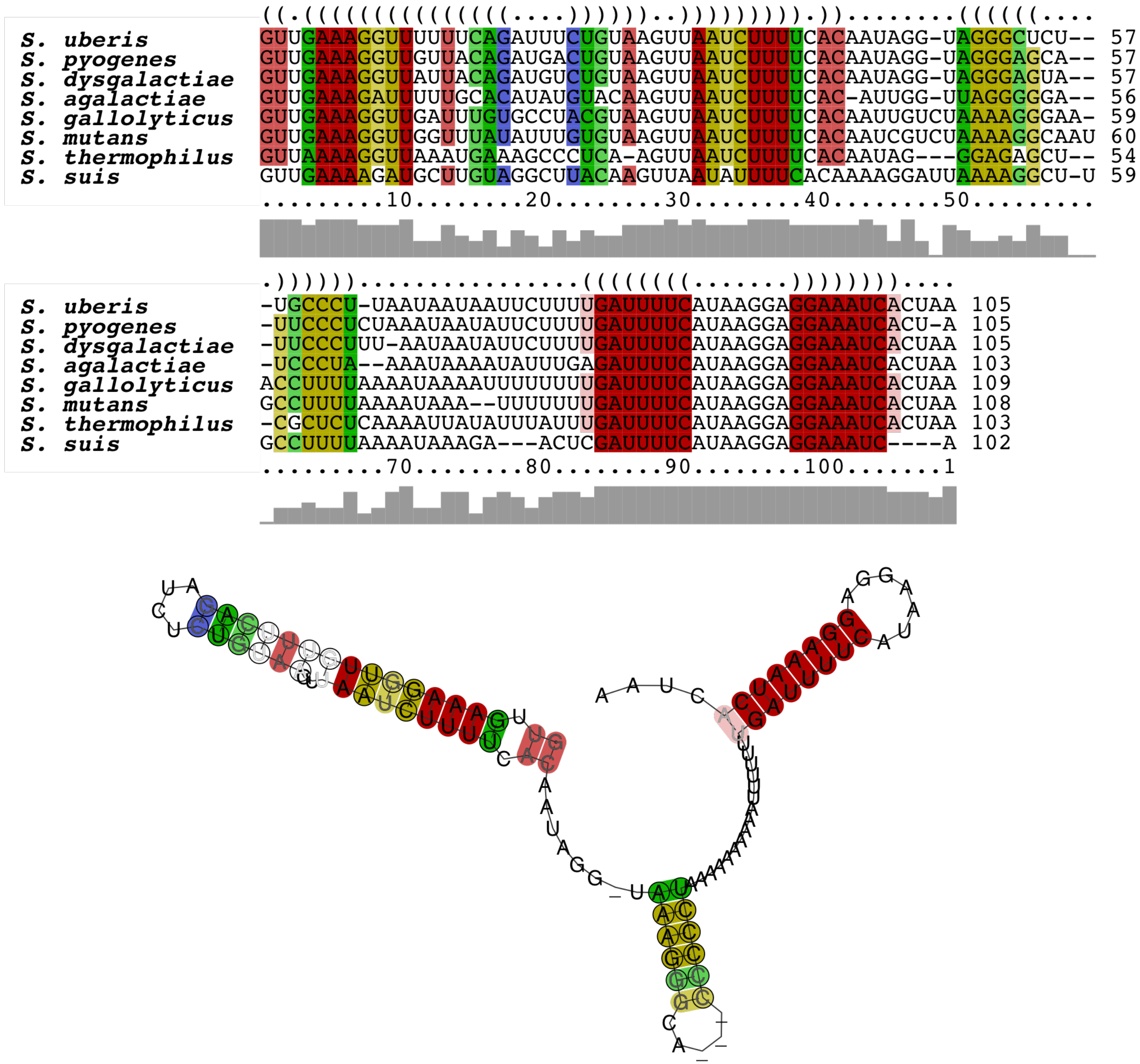
((.((((((((((((((....)))))))..))))))))).))........((((((....
S. uberis GUUGAAAGGUUUUUUCAGAUUUCUGUAAGUUAAUCUUUUCACAAUAGG-UAGGGCUCU-- 57
S. pyogenes GUUGAAAGGUUGUUACAGAUGACUGUAAGUUAAUCUUUUCACAAUAGG-UAGGGAGCA-- 57
S. dysgalactiae GUUGAAAGGUUAUUACAGAUGUCUGUAAGUUAAUCUUUUCACAAUAGG-UAGGGAGUA-- 57
S. agalactiae GUUGAAAGAUUUUUGCACAUAUGUACAAGUUAAUCUUUUCAC-AUUGG-UAGGGGGA-- 56
S. gallolyticus GUUGAAAGGUUGAUUUGUGCCUACGUAAGUUAAUCUUUUCACAAUUGUCUAAAAGGGAA- 59
S. mutans GUUGAAAGGUUGGUUUAUAUUUGUGUAAGUUAAUCUUUUCACAAUCGUCUAAAAGGCAAU 60
S. thermophilus GUUAAAAGGUUAAAUGAAAGCCCUCA-AGUUAAUCUUUUCACAAUAG---GGAGAGCU-- 54
S. suis GUUGAAAAGAUGCUUGUAGGCUUACAAGUUAAUAUUUUCACAAAAGGAUUAAAAGGCU-U 59
.........10........20........30........40........50.........
.))))))................(((((((.......)))))))....
S. uberis -UGCCCU-UAAUAAUAAUUCUUUUGAUUUUCAUAAGGAGGAAAUCACUAA 105
S. pyogenes -UUCCCUCUAAAUAAUAUUCUUUUGAUUUUCAUAAGGAGGAAAUCACU-A 105
S. dysgalactiae -UUCCCUUU-AAUAAUAUUCUUUUGAUUUUCAUAAGGAGGAAAUCACUAA 105
S. agalactiae -UCCCUA--AAAUAAAAUAUUUGAGAUUUUCAUAAGGAGGAAAUCACUAA 103
S. gallolyticus ACCUUUUAAAAUAAAAUUUUUUUUGAUUUUCAUAAGGAGGAAAUCACUAA 109
S. mutans GCCUUUUAAAAUAAA--UUUUUUUGAUUUUCAUAAGGAGGAAAUCACUAA 108
S. thermophilus -CGCUCUCAAAAUUAUAUUUAUUUGAUUUUCAUAAGGAGGAAAUCACUAA 103
S. suis GCCUUUUAAAAUAAAGA---ACUCGAUUUUCAUAAGGAGGAAAUC----A 102
.........70........80........90........100.......1
