# Supplementary material for: Conserved and specific features of Streptococcus pyogenes and Streptococcus agalactiae transcriptional landscapes
Source: BMC Genomics. 2019 Mar 22;20:236. doi: 10.1186/s12864-019-5613-5 (PMC6431027; doi:10.1186/s12864-019-5613-5)

```
                ....(((((((..(((..........)))...)))))))(((((.....(((....)))
S. equi         GUAUGUUUAGUAACUGAUAUCACAAAUAGCCGGCUAAACGAAUACGAAAAUCUAUGAGGA 60
S. uberis       GUAUGUUUAGUAACUGAUAUCAC-AAUAGCCGGCUAAACGAAUACGAAAAUCUAUGAGGA 59
S. mutans       GUAUGUUUAGUAACUGAUCACAU-AAUAGCCGGCUAAACGAAUACGAAAAUCUAUGAGGA 59
S. pyogenes     GUAUGUUUAGUAACUGAUAUCACAAAUAGCCGGCUAAACGAAUAC-AAAAUCUAUGAGGA 59
S. salivarius   GUAUGUUUAGUAACUGAUCA-AA-AAUAGCCGGCUAAACGAAUACGAAAAUCUAUCAGGA 58
S. thermophilus GUAUGUUUAGUAACUGAUCA-AA-AAUAGCCGGCUAAACGAAUACGAAAAUCUAUUAGGA 58
S. gallolyticus GUAUGUUUAGUAACUGAUCA-AA-UAUAGCCGGCUAAACGAAUACGAAAAUCUAUGAGGA 58
S. agalactiae   GUAUGUUUAGUAACUGAUCA-AA--AUAGCCGGCUAAACGAAUACGAAAAUCUAUCAGGA 57
S. suis         GUAUGUUUAGUAACUGAUCA-AA-ACUAGCCGGCUAAACGAAUACAAGAAUCUAAGAGGA 58
S. pneumoniae   GUAUGUUUAGUAACUGAUCACUU-UAUAGCCGGCUAAACGAAUAC-AAAAUCUAUGAGGA 58
                .........10........20........30........40........50.........

                .))))))....
S. equi         GGUAUUC---A 68
S. uberis       GGUAUUC---A 67
S. mutans       GGUAUUCA--A 68
S. pyogenes     GGUAUUCA-CU 69
S. salivarius   GGUAUUCAACG 69
S. thermophilus GGUAUUCAACG 69
S. gallolyticus GGUAUUCAUCG 69
S. agalactiae   GGUAUUCAACG 68
S. suis         GGUAUUCAUCG 69
S. pneumoniae   GGUAUUCAUCG 69
                .........70
```

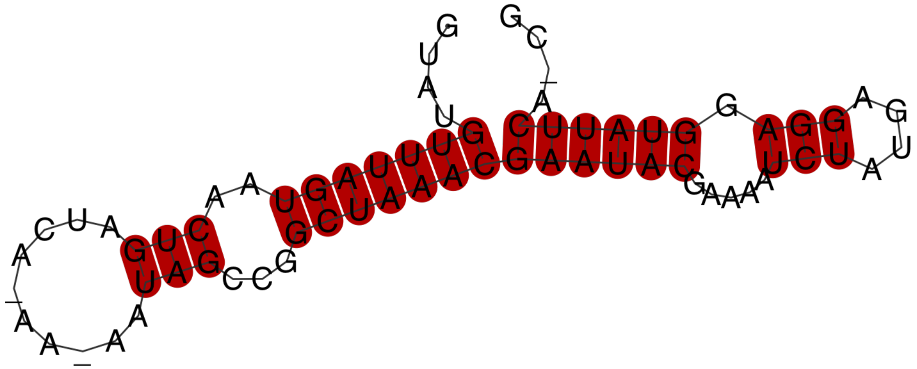

Supplement: Supplementary file 13 — Figure S5. DNA sequence alignment and structure prediction of rpmH 5′ UTR. The DNA sequences of rpmH 5′ UTR in ten streptococci were extracted from NCBI sequence database. The 5′ UTR sequence was predicted by checking for the presence of a potential − 10 box 7–9 nt upstream of the first nucleotide. Alignment and folding prediction were performed by using LocARNA (http://rna.informatik.uni-freiburg.de/LocARNA). Compatible base pairs are colored, where the hue shows the number of different types C-G, G-C, A-U, U-A, G-U or U-G of compatible base pairs in the corresponding columns. The saturation decreases with the number of incompatible base pairs. Accession numbers: S. agalactiae: NC_004368.1; S. gallolyticus: CP013688.1; S. mutans: NC_004350.2; S. pneumoniae: CP016633.2; S. salivarius: CP014144.1; S. suis: NC_012926.1; S. thermophilus: CP016877; S. uberis: NC_012004.1; S. equi: LS483328.1. (PDF 502 kb) [file 12864_2019_5613_MOESM13_ESM.pdf]
